# Metal accumulations in aquatic organisms and health risks in an acid mine-affected site in South China

**DOI:** 10.1007/s10653-021-00923-0

**Published:** 2021-04-19

**Authors:** Wing Sze Chan, Joyanto Routh, Chen Luo, Mårten Dario, Yuqing Miao, Dinggui Luo, Lezhang Wei

**Affiliations:** 1 Department of Thematic Studies - Environmental Change, Linköping University, 58183 Linköping, Sweden; 2 Linköping University - Guangzhou University Research Center on Urban Sustainable Development, Guangzhou University, Guangzhou, 510006 China; 3 School of Geography and Tourism, Anhui Normal University, Wuhu, 241002 China

**Keywords:** Acid mine, Aquatic organisms, Sediments, Metals, Toxicity, Bioaccumulation, Biomagnification

## Abstract

Metal contamination from base metal sulphide mines is a major environmental challenge that poses many ecological and health risks. We examined the metal concentrations in the Dabaoshan mine in South China in water, sediments, and aquatic organisms and their specific characteristics (i.e. size, body tissue, species, and habitat) along the Hengshi and Wengjiang River courses to assess acid mine drainage remediation efforts. Metal concentrations of arsenic, cadmium, chromium, copper, lead, nickel, thallium, and zinc were examined in tissues (i.e. gills, intestines, and muscles) of 17 freshwater species of fish, shrimps, and crabs. Metals in tissues followed the trend: intestines > gills > muscles; nearly all intestine samples exceeded the safe limits of metals analysed in this study. There is a positive correlation between distance from the mine and metal concentrations related to the flow of surface water and the habitat of aquatic organisms. The concentrations of arsenic, copper, and zinc were the highest in aquatic organisms, and the distribution was influenced by physical (distance from mine, currents, and seasonality), chemical (pH and competing ions), and biological (species, habitat, and predator–prey relation) factors. Large demersal fish and benthic fauna had higher concentrations of metals. Bioaccumulation and biomagnification of metals, as well as the high metal pollution index and target hazard quotient (arsenic, cadmium, copper, lead, thallium, and zinc), occurred in bottom feeders (*C. aumtus*, *X. argentea*) and fish belonging to higher trophic levels (*P. fulvidraco*, *O. mossambicus*). Lead and cadmium indicated the highest level of biomagnification from prey to predator. Health risks exist from the dietary intake of common aquatic species such as tilapia and carp besides crustaceans due to high arsenic, cadmium, lead, and thallium levels. Further reduction of metals is necessary to improve the effects of acid mine drainage in the catchment.

**Supplementary Information:**

The online version contains supplementary material available at 10.1007/s10653-021-00923-0.

## Introduction

Mining of sulphidic ores has caused severe environmental problems, especially in soil, surface and groundwater bodies from acid mine drainage (AMD) (Luo et al., 2020; Shu et al., 2018; Zhao et al., 2012a, 2012b). AMD results in elevated levels of metals, and as a result, extensive remediation is needed to reverse these changes (Zhuang et al., 2013; Dudka and Adraiano, 1997). High concentrations of very toxic metals released because of the acidic conditions, weathering of mine tailings, and exposed mineral wastes stored near the ore processing plants pose a significant threat to human health (Liao et al., 2016; Cai et al., 2015; Liu et al., 2010, Zhou et al., 2007; Gnandi et al., 2006; Li et al., 2006). As one of the largest suppliers of metalliferous ores and non-metallic minerals, China has around 230,000 mines that produce millions of tons of mine wastes every year (Zhuang et al., 2014). The Guangdong province in SE China is one of the largest centres for mining in the country. The rapid expansion in mining and processing of metal ores have caused major environmental concerns due to AMD and widespread metal contamination in soils, fluvial sediments, surface and groundwater near the mines (Shu et al., 2018; Liao et al., 2016).

Aquatic organisms, including fish, crustaceans, and mollusks, can accumulate metals in their bodies. These contaminants can potentially magnify within the food chain to a level that can be highly toxic to humans (Qiu et al., 2011; Telisman et al., 2000). These organisms are referred to as biomonitors, and numerous investigations have been carried out worldwide to trace metal contamination and toxicity studies in them (Lebepe et al., 2020; Qiu et al., 2011; Schmitt et al., 2007; Brumbaugh et al., 2005). For instance, fish can accumulate metals in their body by consuming contaminated sediments and prey and direct absorption via their gills (Chen & Chen, 1999). The uptake of metals is a major concern because fish is a common food in the human diet and an essential nutrient source.

The Guangdong province consists of many enriched mineral deposits like ferrous and non-ferrous metals, rare earth metals, and radioactive elements in host rocks (Zhou et al., 2007). This has resulted in a flourishing mining industry that is an important and crucial contribution to the regional economy and growth. However, mining has also led to numerous environmental concerns. Leaching from mine tailings, weathering, and run-off of mine wastes have spread metals further away from the mines exacerbating the contamination problem beyond the limits of the mines and their subsidies (Luo et al., 2020; Shu et al., 2018; Liao et al., 2016; Zhao et al., 2012a, 2012b; Zhou et al., 2007). The current study focuses on one of the largest base metal mines in the Guangdong province—the Dabaoshan mine site (DMS), which has been operational for several decades and faces AMD problems. Previous studies at DMS indicated that arsenic, cadmium, copper, lead, thallium, and zinc are higher than the national safety level in the water and surrounding soil and sediments because of naturally high concentrations. The high concentrations are further exacerbated by the disposal of mine waste and tailings near the mine site (Luo et al., 2020; Shu et al., 2018; Liao et al., 2016; Zhuang et al., 2014, Zhuang et al., 2013; Zhao et al., 2012a, 2012b; Zhuang et al., 2009; Zhou et al., 2007). Nearly 83 villages, 585 × 10^4^ m^2^ of paddy fields, and 21 × 10^4^ m^2^ ponds have been affected by AMD (Shu et al., 2016). Hence, both on-site and off-site remediation methods have been implemented at DMS in recent years in terms of liming, on-site wastewater treatment generated from ore processing, storage, revegetation, and management of mine tailings to lower the mobilization of metals (Zhao et al., 2012a, 2012b). Luo et al. (2020) investigated the effects of ongoing remediation methods on metal dispersion and its current levels in the surroundings. The detailed analyses of metal (arsenic, cadmium, copper, lead, and zinc) concentrations in river water, groundwater, sediments, and soils were used to categorize the sampling sites into potentially low to high ecological risk zones (Luo et al., 2020).

**Fig. 1:**
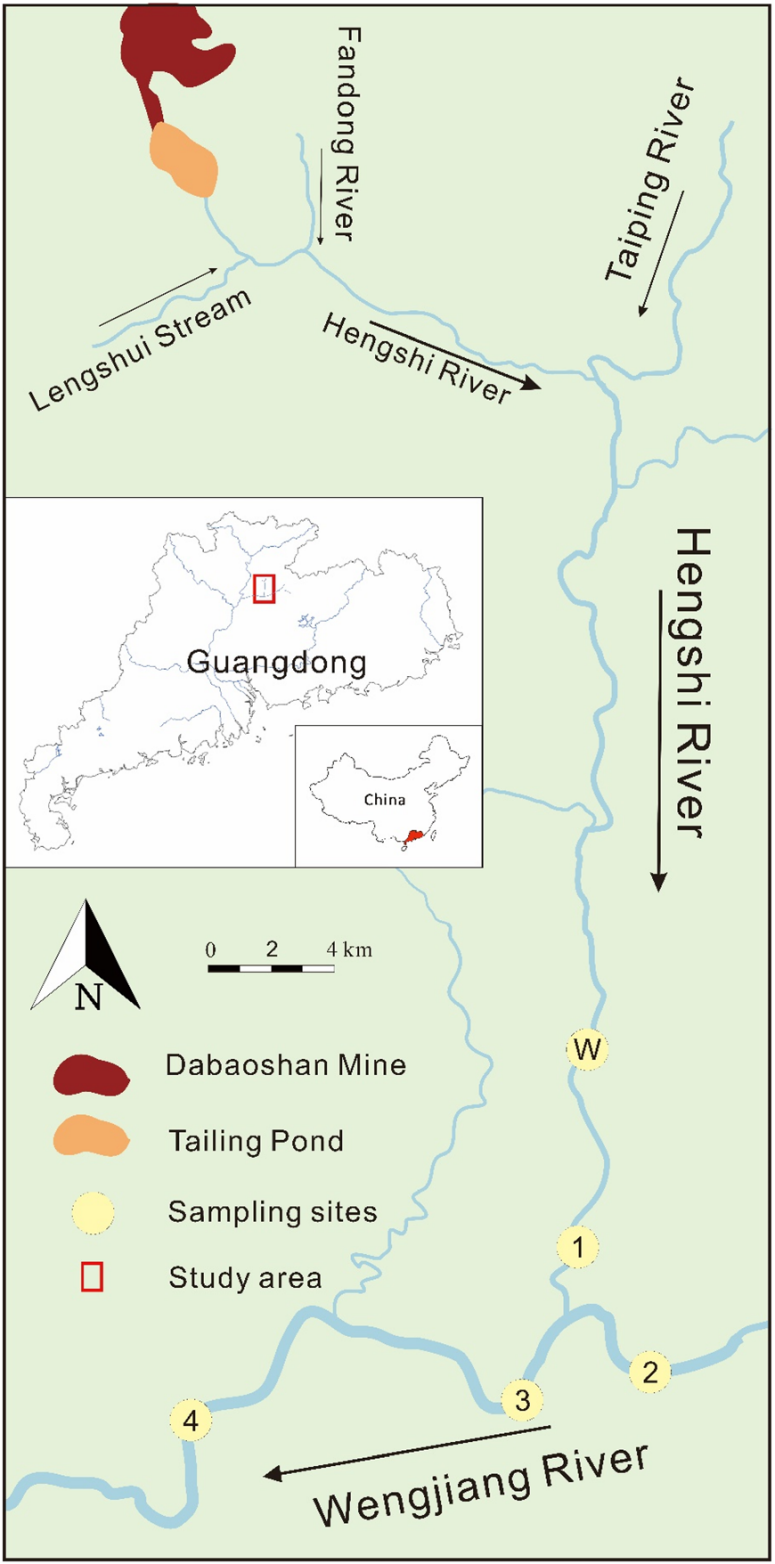
Sampling sites in the Hengshi and Wengjiang Rivers near Dabaoshan mine, Guangdong province, China

Where long-term monitoring data are absent, geochemical and mineralogical characterization of metals in sediments, surface water, and aquatic organisms around mine sites can provide useful information about assessing AMD influence and predicting its future impacts. To the best of our knowledge, not much information exists regarding the status of toxic metals in flora and fauna and if it poses potential risks to people around the Dabaoshan mine. Hence, the current study evaluates the accumulation of metals in aquatic organisms following the assessment made by Luo et al. (2020) about the state of AMD remediation initiatives in sediments and surface water bodies around DMS. To evaluate the different pathways for metal intake and accumulation in aquatic organisms (e.g. fish, shrimps, crabs, and snails), samples retrieved from the Hengshi and Wengjiang rivers downstream from DMS were investigated (Fig. 1). Moreover, water and fluvial sediments were also collected from the same sites where aquatic organisms were caught to explain metal contamination in the aquatic habitat. The specific aims are to investigate the: (1) concentration of metals in aquatic organisms in tissues including gills, intestines, muscles, and heads (only in case of shrimps), (2) relationship between metal concentrations in surface water and sediments versus different aquatic organisms, (3) differences in metal concentrations in relation to the size of other aquatic organisms, their habitats, and position in a telescoping food chain, and (4) bioaccumulation and biomagnification levels in various aquatic organisms in the Hengshi and Wengjiang Rivers to assess the potential health risks. The data generated will be evaluated to determine the safety of the fresh catch from these rivers that are eaten or sold in the local and provincial markets. Tracing the pathway of metals transferring from water and sediments into aquatic organisms and then to humans and factors that influence this process can provide vital information. The knowledge can be used to initiate remediation plans for sustainable mining practices that produce fewer contaminants and adhere to safe on-site remediation or disposal practices (Dixit et al., 2015; Wuana & Okieimen, 2011).

## Materials and methods

### Study area

The Dabaoshan mine (24° 31′ 37″ N, 113° 42′ 49″ E) is positioned in the south-east of Shaoguan in Guangdong province, China (Fig. 1). Dabaoshan is situated in a sub-tropical region with high humidity and intense monsoon spells. The average temperature is 20 °C with annual precipitation of 1800 mm (Zhao et al., 2012a). The site has been intermittently mined since the Song dynasty (960–1279 CE; Zhao et al., 2012a, 2012b). Intensive mining of copper and iron ore at the mine started in the 1970s, but it has slowed down in recent years. Large amounts of mine tailings containing pyrite, pyrrhotite, chalcopyrite, sphalerite, and galena are left exposed subject to rapid oxidation and release of metals (Ma et al., 2011). The Hengshi River flowing by the mine site originates in limestone terrain, and pH in the water was >8 before it joins the AMD-affected area downstream (Ma et al., 2011). A dam was built to hold the treated AMD water, and the treated water is then released into the Hengshi River. Iron-bearing minerals as goethite and hematite have deposited in the acidic (average pH < 4.9) waters downstream, resulting in the characteristic brown colour in the river course immediately after the mine. The upstream section of the Hengshi River (after the mine) has high concentrations of many metals (see Luo et al., 2020). Also, in this section, the water level is shallow, and it has low pH (2.8–5.7; Luo et al., 2020) for aquatic organisms to thrive. A survey in the upstream section of the river in January 2019 indicated the absence of fish, shrimps, crabs, and snails that are more abundant in the downstream sections. Many small villages and farmland are located along the upper reaches of the Taiping and Hengshi Rivers. The land-use pattern indicates mainly forested and hilly areas around DMS, which changes to widespread agriculture and residential areas beyond the Shangba village (Zhao et al., 2012a, 2012b). Agricultural land has mainly developed in the alluvial soils along the river course downstream.

### Sample collection

The study focused on the spatiotemporal analysis of metal concentrations. The sampling sites covered the lower reaches of the Hengshi River until it joined the Wengjiang River and all the way downstream to the spot where the two river systems confluence (Fig. 1). The closest sampling site to the mine (site W) and the farthest site (site 4) from the tailings were approximately 32 km and 54 km from the discharge point, respectively. As part of the preliminary screening process, fish samples were first collected in January 2019 at site W, coinciding with water and sediments samples retrieved along the Hengshi River (see details in Luo et al., 2020). Site W in the Hengshi River was the first spot along the river course where we noted fish being caught by local fishermen. The water level was deeper (2–3 m), and the pH was near neutral. In June 2019 (during the monsoon season), the sampling event was expanded to include sites 1–4, and simultaneously, sediment cores and water samples were also collected from these locations. Site 1 and site W were both located at the lower reaches of the Hengshi River, while sites 3 and 4 were from the middle and lower reaches of the Wengjiang river after the two river systems confluence. Site 2 was in the upstream section of the larger Wengjiang river, and it did not receive any direct discharge from DMS or the polluted Hengshi river. The water depth was 2–3 m in winter and 3–4 m in summer downstream from site W.

The fishing nets (1.5 m * 100 m with 3.33 cm grid size) were spread at the sites vertically in the direction of the current in the early morning hours. The different aquatic organisms that were caught in the nets within the next 12 h were sampled. The different aquatic organisms retrieved included 62 fishes, 18 shrimps, 5 crabs, and 10 mollusks. The samples were removed from the nets, euthanized following established protocols, sealed in polyethylene bags, and refrigerated. When multiple samples of the same species were caught in the fishing net, only a few samples representative of the catch, and that would provide us enough material for all analyses, were saved while the remaining catch was released. Surface sediments from the river (0–4 cm) were collected using a gravity corer and sealed in polyethylene bags. The aquatic organisms were kept refrigerated and transported to the laboratory, where they were dissected within 24 h of reaching the Guangzhou University laboratory. The tissue samples were stored at −20 °C and sediments at −4 °C until further treatment.

### Sample pre-treatment and analyses

The water samples were acidified with concentrated HNO_3_ in the field. The sediment samples were freeze-dried, lightly crushed with a mortar and pestle, and sieved (2 mm). The aquatic organisms were measured for their length and width, weighed, and then dissected with stainless steel scalpels and knives to extract the muscles and gills. In the larger species, the intestine was also sampled to represent the gut content. In the case of shrimps, heads, as well as the muscles, were collected. Only muscles were sampled in crabs, while intestines and muscles were sampled in few snails caught in the fishing nets. Two to three samples of the same species were dissected if there were multiple samples of the same type at a particular site. The different species of fishes were identified by physical examination followed by taxonomic classification by an ichthyologist. Their habitat, predator–prey relationships, and trophic status were established from the literature and online database FishBase (Froese & Pauly, 2019). The shrimps, crabs, and snails were not identified at the species level. After dissection, the tissues were stored in polyethylene tubes and freeze-dried. No efforts were made to separate bones from tissues, and the freeze-dried material was crushed into powder using a coffee blender. Water samples were collected and analysed earlier (see Luo et al., 2020), and the results were referred to provide a broader analysis of metal pollution and its impacts.

### Chemical analysis

Digestion of sediment samples involved an autoclave for extracting metals. 0.5 g of the freeze-dried crushed sample was weighed, and 20 ml of 7 M HNO_3_ was added to it (SIS, 1993). Blank and standard reference materials (Jet Rock and PACS-2) were also digested in the same way during the extraction for QA/QC purposes. The samples were put in an autoclave for 30 min at 120 °C. After that, the samples were cooled at room temperature, filtered, and diluted to 50 ml using deionized water.

Microwave-assisted metal digestion was used for the tissue samples. 0.3 g of the freeze-dried crushed sample was weighed and digested with a mixture of 8 ml of HNO_3_ and 2 ml of H_2_O_2_. There were blanks, and standard reference material (GBW 08573) used during each batch of digestion to verify the digestion output and QA/QC. The samples were placed in a microwave oven (Ethos, Milestone) and heated to 180 °C for 30 min, and the temperature was maintained at 180 °C for 150 min. The samples were cooled at room temperature, filtered, and diluted with 50 ml of deionized water for metal analyses. An inductively coupled plasma mass spectrometer (PerkinElmer NexION 300D) was used to analyse various metals (arsenic, cadmium, chromium, copper, lead, nickel, thallium, and zinc) in the acidified water and digested samples. These metals were selected due to their toxic effects in aquatic ecosystems or their high levels near the mine in previous studies (Luo et al., 2020; Shu et al., 2018; Liao et al., 2016; Zhao et al., 2012a, 2012b; Zhou et al., 2007).

### Risk analysis

The bioaccumulation factor (BAF) in organisms was calculated according to Barron (2003):

1 $${\text{BAF}} = \frac{Ct}{{Cs}}$$

*Ct* = metal concentration (in mg/kg dry weight) in tissue; *Cs* = metal concentration (in mg/kg dry weight) in sediment. BAF indicates bioaccumulation in gills, intestines, and muscles in different aquatic organisms. A higher value of BAF means a higher level of bioaccumulation of metals.

The biomagnification factor (BMF) in organisms was calculated according to Romero and Keith (2012):

2 $${\text{BMF}} = \frac{Cb}{{Cd}}$$

*Cb* = metal concentration (in mg/kg dry weight) in the predator; *Cd* = metal concentration (in mg/kg dry weight) in the prey. A higher value of BMF indicates a higher degree of biomagnification of metals in the food chain. Metal concentrations in the muscles of the preys were used for the calculations.

The predator–prey relationship in the water body was not investigated because it involves far more rigor in sampling, observations, and measurement of different ecological parameters (Taylor, 2004). However, such information and inference were drawn from the online database FishBase (Froese & Pauly, 2019). The database included information on similar aquatic species, habitat, and food habits and provided the necessary cues for understanding such relationships and cross-references.

The target hazard quotient (THQ) of metals for fish was calculated using the following formula (Ahmed et al., 2015; Chien et al., 2002):

3 $${\text{THQ}} = \frac{{{\text{EF}} \times {\text{ED}} \times {\text{FI}} \times {\text{MC}}}}{{{\text{RfDo}} \times {\text{BW}} \times {\text{AT}}}} \times 10^{ - 3}$$

EF = exposure frequency (365 days/year); ED = exposure duration (70 years); FI = fish ingestion (in grams per person per day), which is estimated to be 33.5 g per person per day according to (Zhuang et al., 2013); MC = metal concentration in fish (in mg/kg dry weight); RfDo = oral reference dose (in mg/kg/day); BW = average adult body weight (60 kg); AT = averaging time for non-carcinogens (365 days/year × number of exposure years, assuming 70 years). Oral reference doses were based on 3 × 10^–4^ for arsenic; 1 × 10^–3^ for cadmium; 4 × 10^–2^ for copper; 3 × 10^–3^ for chromium; 2 × 10^–2^ for nickel; 4 × 10^–3^ for lead; 1 × 10^–5^ for thallium; 3 × 10^–1^ for zinc in mg/kg/day (USEPA, 2012). THQ < 1 suggests adverse health effects are not probable, while THQ ≥ 1 suggests a high probability of adverse health effects.

The metal pollution index (MPI) measures the total accumulation of metals in tissues and different sampling sites investigated in this study. It was calculated according to Usero et al. (1997):

4 $${\text{MPI }} = \left( {C1 \times C2 \times C3 \times \cdots Cn} \right)^{1/n}$$

*Cn* = concentration for the metal “*n*” in the sample in mg/kg dry weight. A higher MPI implies a higher total metal content and metal accumulations in the samples.

### Statistical analysis

Principal component analysis (PCA) was used to examine the multi-dimensional data. The analysis was done using Minitab 19.2020.1. A plot of the first two principal components that grouped all the aquatic organisms was evaluated. Further, the hierarchical cluster analysis (HCA) was used to measure the distance between each group of aquatic organisms in terms of their metal concentrations and then group the samples that were close together. The clustering was performed using SPSS Statistics V26. Ward’s method was used to generate the dendrogram (Ward, 1963).

## Results

### Metals in water and sediments

Water chemistry in surface water samples from the sites is indicated in Table 1. In general, conductivity and elemental concentrations were high at site 1 (e.g. copper, lead, and zinc). pH levels were near neutral, and concentrations of major elements as calcium and iron were high in the upstream section due to AMD and liming. The sediment samples had higher concentrations of metals at site 1, closer to DMS, than sites 2, 3, and 4 (Table 2). Lead and arsenic exhibited more than double the concentration at site 3 compared to site 1. On the other hand, site 2 displayed the lowest concentration of metals except for chromium and nickel. Almost all the samples from site 2 met the levels specified by the Chinese Marine Sediment Primary Standard, whereas most of the samples from sites 1, 3, and 4 exceeded the specified safe limits. Copper, lead, and arsenic levels were high in bottom sediments in sites 1, 3, and 4. For site W, the levels of cadmium, copper, and zinc were high. Metal concentrations in terms of abundance at the sampling sites were as follows: Zn > Cu > Pb > As > Cr > Cd. In terms of the abundance of these metals with respect to the Chinese standard, the distribution followed the order: Cu > As > Pb > Cd > Zn > Cr; thallium was not listed in the Chinese Marine Sediment Primary Standard and was not compared.

**Table 1:** Metal concentrations from the Hengshi and Wengjiang Rivers near Dabaoshan mine, China, located close to where fish were caught

Sites	Temp (°C)	pH	EC (μs/cm)	As (μg/L)	Cd (μg/L)	Cu (μg/L)	Mn (μg/L)	Pb (μg/L)	Tl (μg/L)	Zn (μg/L)	Ca (mg/L)	Fe (μg/L)	K (mg/L)	Mg (mg/L)	Na (mg/L)
1	25.5	6.73	479	3.10	7.65	275	1983	27.6	0.11	1363	70.4	7612	1.48	8.58	1.75
2	26.1	6.60	134	2.49	3.74	128	807	8.38	0.10	659	13.8	1790	1.60	3.38	1.11
3	25.7	6.96	148	2.20	0.48	12.1	195	4.92	0.10	58.7	16.8	791	2.52	2.07	2.76
4	25.7	7.37	189	4.26	1.46	61.1	496	17.8	0.11	231	25.4	2740	2.49	2.89	2.24
W	17.2	7.30	369	1.33	0.15	4.23	39.6	0.80	0.03	45.2	64.7	87.2	2.36	11.7	4.56

**Table 2:** Metal concentrations (mg/kg dry weight) in fluvial sediments collected from the Hengshi and Wengjiang Rivers near Dabaoshan mine, China

Site	Sample depth (cm)^a^	As	Cd	Cr	Cu	Ni	Pb	Tl	Zn
Site 1	0–2	81.0	7.00	23.0	1464	26.0	275	0.40	1841
	2–4	80.0	6.80	23.0	1499	27.0	278	0.41	1812
Site 2	0–2	17.0	0.49	17.0	39.0	15.0	20.0	0.24	66.0
	2–4	18.0	0.43	17.0	35.0	15.0	20.0	0.24	66.0
Site 3	0–2	206	2.80	25.0	856	30.0	595	0.40	851
	2–4	268	3.50	31.0	1047	35.0	745	0.48	1048
Site 4	0–2	63.8	2.22	–	199	–	114	0.46	321
	2–4	63.6	2.47	–	213	–	118	0.46	347
Luo et al. (2020) (Site W)		19.0	1.42	4.10	118	4.80	45.9	0.06	246
Measured values for certified reference materials (certified values in parenthesis)									
JR-1		18.7 (16.5)	1.56 (2.15)	81.3 (90)	104 (102)	81.3 (90.0)	15.3 (20.0)	1.72 (–)	175 (166)
PACS-II		25.7 (26.2)	2.40 (2.11)	82.6 (90.7)	297 (310)	82.6 (90.7)	169 (183)	0.43 (0.60)	351 (364)
Chinese safety standard for metal concentrations in sediments									
China State Bureau of Quality and Technical Supervision (2002)		20.0	0.50	80.0	35.0	16.0	60.0	Not Stated	150

^a^Two samples from each depth were run, and the average value was reported

JR-1: Jet Rock 1; PACS-11 (Certified standard, reference sediment)

### Metals in aquatic organisms

Metal concentrations in the aquatic organisms from different sites along the Hengshi and Wengjiang Rivers are indicated in the Supplementary data (Tables S1, S2, S3, and S4). We compared the values in different tissues (gills, muscles, and intestines) to the limits proposed by the different international standards (Supplementary data Table S.5) to assess contamination levels in aquatic organisms at these sites along the river courses.

*Arsenic* For arsenic, all the muscles, gills, and shrimp heads met the Chinese standard (2.5 mg/kg) and were less than half the concentration noted in intestine samples. The highest level of arsenic (32.0 mg/kg) was found in the intestines of *P. fulvidraco* collected from site W, which was almost 13 times that of the Chinese standard (Supplementary data Tables S1–S4).

*Cadmium* Almost none of the intestine and shrimp heads met the standard specified for cadmium, except the intestine tissues of *S. chuatsi* from site W, which had a significantly low concentration (0.08 mg/kg). Although the UNEP standard has the highest permissible limit for cadmium in aquatic organisms (1.5 mg/kg) only <54% of the samples were below the stated limit. The highest cadmium concentration was found in the intestine of snails from site 4, with around 57.0 mg/kg (Supplementary data Tables S1–S4), which is almost 230 times the international standards, namely TFC, EC, and FAO limits. Muscle tissue in *C. aumtus* from site 1 and muscle tissue in *S. chuatsi* from site W had a very low cadmium concentration or below the detection level (Supplementary data Tables S1–S4).

*Chromium* Around 32% of the samples failed to meet the Chinese standard limits (1.00 mg/kg) for chromium, whereas about 12% did not meet the IAEA-407 standard (3.65 mg/kg); all these were intestine samples. The highest level of chromium was 29.0 mg/kg (Supplementary data Tables S1–S4), which was over 28 times the Chinese standard and was found in the intestine of *X. argentea* from site 1. The lowest chromium level was found in the muscles of *O. mossambicus* from site W (0.16 mg/kg; Supplementary data Tables S1–S4).

*Copper* Only 42% of the samples met the IAEA-407 standard (16.4 mg/kg), whereas nearly 20% of the samples did not meet the FAO standard (150 mg/kg), which is the international standard with the highest value. The sample containing the highest concentration of copper occurred in the intestines of *O. mossambicus* from site 2, containing 1346 mg/kg of copper (Supplementary data Tables S1–S4), which equalled 82 times the IAEA-407 and 9 times the FAO standard.

*Lead* In the international standards, lead in aquatic organisms varied from 0.60 to 2.50 mg/kg. Notably, about 28% of the samples in this study did not meet the limit specified by the IAEA (0.60 kg/mg), and about 14% were above the Chinese and FAO standards (2.5 mg/kg). Almost all the gills and muscles met the permissible limit for lead specified by the international standards; the only exception was the gills in *A. nobilis* (4.10 mg/kg) (Supplementary data Tables S1–S4). The highest lead concentration was 93.0 mg/kg in the intestine tissue in *O. mossambicus* at site 2, which was close to 155 times the IAEA standard; this was much higher than the intestines in *S. chuatsi* from site W, which contained the lowest level of lead (Supplementary data Tables S1–S4).

*Nickel* Nearly 17% of the samples exceeded the IAEA-407 limit (3.00 mg/kg) for nickel, and almost 12% of samples were beyond the Chinese national standard (5.00 mg/kg). The sample that contained the highest nickel level (42.0 mg/kg) occurred at site W in the intestine of *X. argentea* (Supplementary data Tables S1–S4) is almost 14 times the IAEA-407 standard followed by the intestine tissue from the same species in site 1. In contrast, the lowest nickel concentration was in the muscles of *S. chuatsi* and *C. carpio* from site W (Supplementary data Tables S1–S4).

*Thallium *Nearly 12% of samples exceeded the permissible value as indicated in the EPA standard (0.25 mg/kg). The highest concentration of thallium occurred in the intestine of *X. argentea* from site 1 (0.83 mg/kg), which is more than 3 times the value in the standard; the lowest concentration occurred in site 1, in the muscle tissue of *C. aumtus* (0.004 mg/kg) (Supplementary data Tables S1–S4).

*Zinc *In 47% of the samples, zinc did not meet the Chinese standard (100 mg/kg), while about 26% of the samples were above the FAO standard (200 mg/kg). The highest concentration was found in the intestines of snails collected from site 4 (4981 mg/kg) (Supplementary data Tables S1–S4), which was nearly 50 times the Chinese standard.

**Fig. 2:**
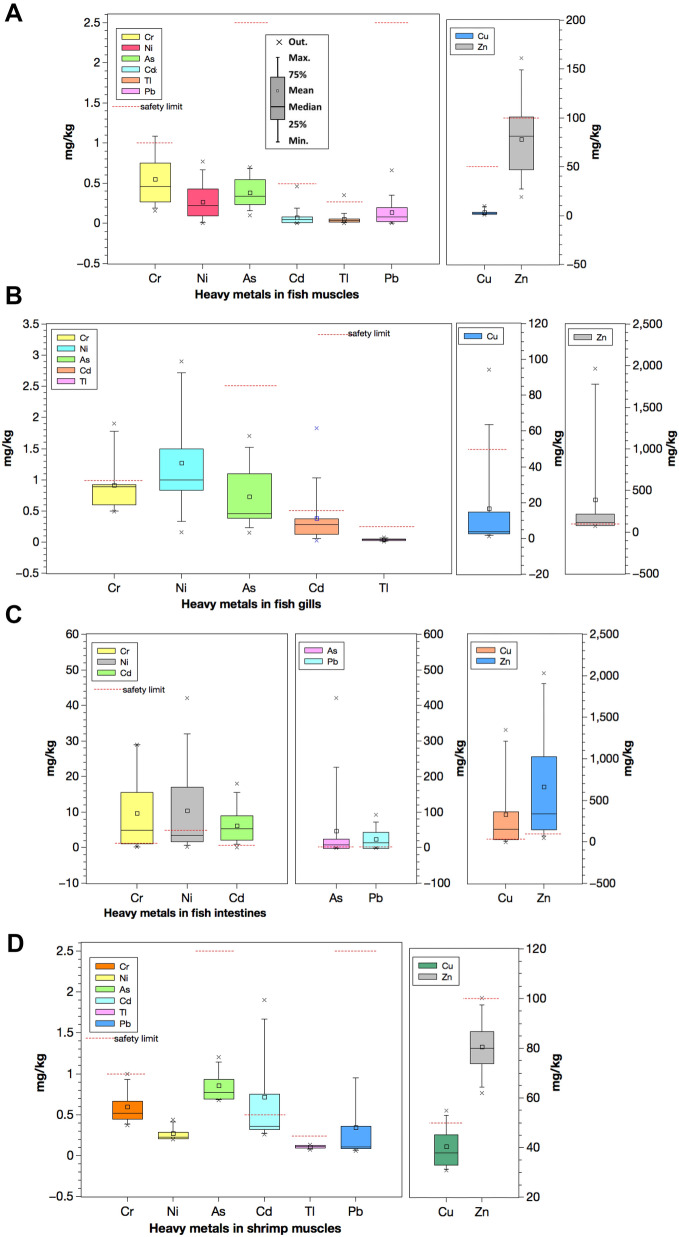

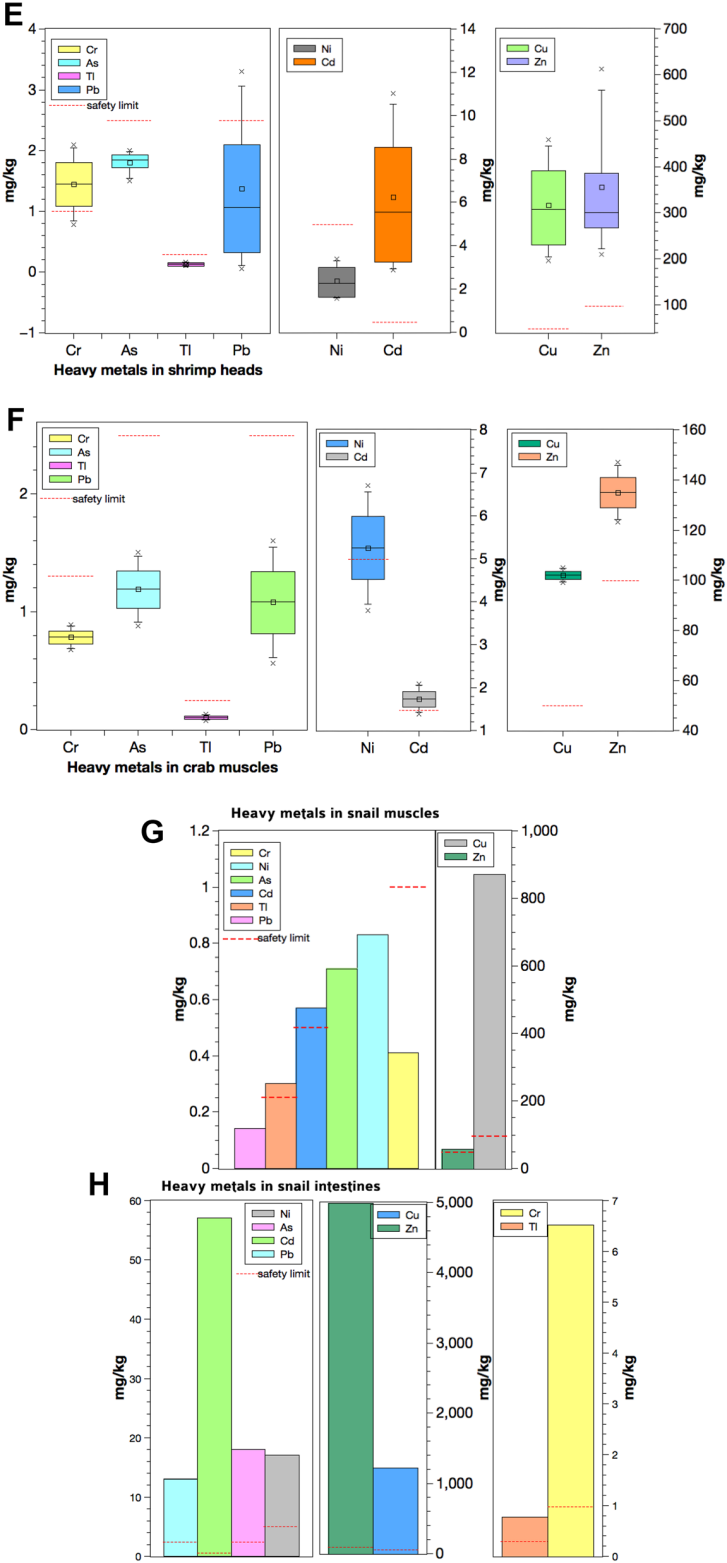
Metals in aquatic organisms from the Hengshi and Wengjiang Rivers near Dabaoshan mine, China. The Chinese safety limit (Cr, As, Cd, Pb, Ni, Cu, and Zn) and EPA safety limit (Tl) are also indicated. Metal concentrations (mg/kg) in: (**a**) fish muscles, (**b**) fish gills, (**c**) fish intestines, (**d**) shrimp muscles, (**e**) shrimp heads, (**f**) crab muscles, (**g**) snail muscles, and (**h**) snail intestines (snails were only found in one site unlike the other samples that were caught from multiple locations). The histograms indicate maximum, minimum, mean, median, and mode values

Metals in different fish tissues indicated the following trend: intestines > gills > muscles. For shrimps, the head contained a higher concentration of metals than its muscles. Similarly, snails also had a high level of metals in their intestines than muscles. All the sites indicated high concentrations of metals in most intestine samples (Fig. 2). Notably, site W had the lowest concentration of metals in most cases. Among all the muscle samples, crabs contained the highest metal concentrations, especially arsenic, cadmium, copper, nickel, and lead. For gills, *A. nobilis* from site 4 comprised the highest concentrations of several metals: arsenic, chromium, nickel, and lead. Lastly, the intestine tissues in *X. argentea* from site 1 and *O. mossambicus* from site 2 had high concentrations of metals, particularly chromium, copper, lead, nickel, and thallium. *S. chuatsi* from site W had a low concentration of metals specifically—arsenic, copper, nickel, lead, and zinc. The metal concentrations indicated the following trend when arranged according to the number of samples that did not meet the corresponding permissible limits: As > Zn > Tl > Cr > Cd > Cu > Ni > Pb. This trend was almost like the one where the degree that the metal concentrations in samples exceeded the corresponding safety standard: As > Zn > Cu > Cd > Tl > Ni > Cr > Pb.

### Principal component analysis

Principal component analysis (PCA) was used to identify the correlation between different fish tissues and metal concentrations theoretically. Only fish samples were analysed as the sample numbers of shrimps, crabs, and snails were not large enough for PCA. The eigenvalues described the variance explained by each component; the eigenvalue decreased with an increase in the number of principal components (PCs). Only the first two PCs were considered as 76.8% of the variation was captured by them. The first and second PCs explained 57.8% and 19% of the total variance, respectively.

**Fig. 3:**
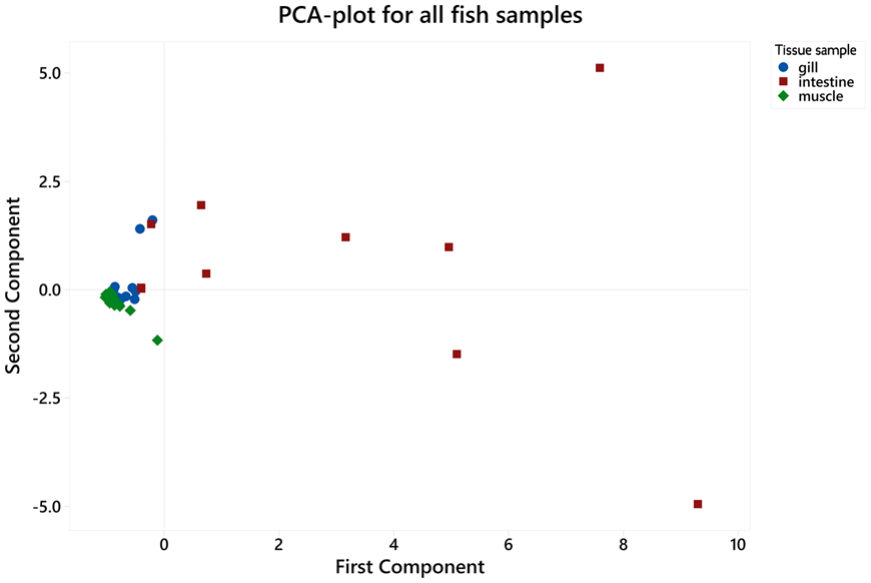
Principal component analysis plot for all the fish samples grouped by their parts (gill, head, intestine, and muscle) near Dabaoshan mine, China

According to the PCA plot (Fig. 3), it was observed that nearly all intestine samples were separated from the gills and muscles by the first PC. However, the intestine samples did not cluster closely with each other, suggesting a considerable dissimilarity between them. The five intestine samples in the centre were regarded as one cluster with a high loading for the first PC. Most of the muscle and gill samples clustered together with a negative loading for the first PC, while two samples of gills were separated by the second PC even though the difference was not considered significant.

### Hierarchical clustering analysis

Hierarchical cluster analysis (HCA) was used to establish a hierarchy of clusters because it grouped the subjects with similar features into clusters to better study the differences in metal concentrations and their accumulation in the aquatic organisms. In this study, three large clusters were distinct in the dendrogram (see supplementary data; Figure S.1). The first layer of the dendrogram (Figure S.1) categorized the three most obvious clusters. The biggest one covered almost all the muscles and gills; the second cluster included the gills and intestines of *C. carpio*; the third one comprised of shrimp heads, together with the muscles of snails and *P. fulvidraco* and the intestine in *C. aumtus*. The clusters indicated that most of the gills and muscles had similar metal concentrations. The intestine samples in *P. fulvidraco*, *O. mossambicus*, *X. argentea* and those in snails had large differences in metal concentrations. The results suggested that the metal concentrations in shrimp heads were like the gills and muscles instead of intestines. There were a few intestine samples belonging to *S. chuatsi, A. macropterus,* and *H. maculatus*, which grouped with the cluster for gills and muscles, meaning that their metal concentrations were similar in contrast to other intestine samples.

### Bioaccumulation of metals

According to Rashed (2001), bioaccumulation of toxic compounds in samples is indicated when BAF is >1. The higher the BAF, the higher is the bioaccumulation level. The preliminary study assesses metal concentrations and if it poses a risk. The BAFs indicated that >15% of the values were >1, implying bioaccumulation of metals occurred in many aquatic organisms examined in this study. In most cases, bioaccumulation occurred in the intestines and gills. Only in few cases, bioaccumulation occurred in muscles, as in *P. altivelis, H. bleekeri, C. aumtus, O. mossambicus,* snails, and shrimps. However, none of the BAF values in muscle samples were >2.0 except for *O. mossambicus*, meaning that bioaccumulation in muscle tissues was not as high as noted in gills and intestines. The highest BAF value was in chromium and nickel in the intestines of *C. aumtus* from site W. The value was also high compared to all the other samples or sites.

The bioaccumulation of chromium and nickel in intestines and gills was high in some species from site W, such as in *A. macropterus* and *P. fulvidraco.* Similarly, cadmium, copper, and zinc in the muscles of *O. mossambicus* from site W reached 39.9, 36.6, and 27.0, respectively, which were very high levels of bioaccumulation. Bioaccumulation of cadmium and zinc in the intestines of snails was very high, i.e. 24 and 15, respectively. Additionally, *C. carpio* from site W was found to have high BAF for zinc in its gills and intestines, and *P. fulvidraco* from site W for cadmium in its intestines. *X. argentea,* which had relatively high metal concentrations, particularly in intestines, did not indicate a high BAF. In general, samples from site W had a higher value of BAF. The BAF for metals showed the order Cr > Ni > Cd > Cu > Zn > Tl > Pb > As. Notably, zinc indicated bioaccumulation in the largest number of samples.

### Biomagnification of metals

The calculation of BMF in aquatic organisms is based on some simple assumptions: (1) dietary consumption is the primary source of metal exposure, (2) the predator–prey relationship is simple, and (3) predators consume the prey completely. The BMFs in carnivores in this study are listed in supplementary data Table S.7. The BMFs were calculated using the corresponding prey(s) of the carnivores. Biomagnification of metals occurs when BMF is >1 (Romero & Keith, 2012); the higher the BMF, the higher is the level of biomagnification. The result of BMFs showed that >30% of the values were >1, implying biomagnification of several metals in the aquatic organisms investigated in this study. Lead and cadmium had the highest biomagnification level from prey to predator. In contrast, copper had the least sign of biomagnification in the food chain.

The highest level of biomagnification was observed in *P. fulvidraco* and *C. aumtus*, which showed a BMF of 644 (lead), 464 (cadmium), 109 (arsenic), 48 (copper), 25 (nickel), 19 (chromium), 14 (thallium), and 4.2 (zinc). *P. fulvidraco* indicated the highest level of biomagnification in the study. The fish had a high BMF for prey for most metals, particularly in lead, arsenic, and cadmium. Besides, both *P. fulvidraco* and *H. maculatus* indicated high biomagnification in their intestines. In the case of *S. chuatsi*, high biomagnification occurred in gills. Besides, *S. chuatsi* showed a high level of biomagnification for nickel, a moderate level of biomagnification for thallium, lead, chromium and cadmium, and no sign of biomagnification for arsenic and copper. The intestines in *S. chuatsi* did not show any biomagnification except for lead (prey 6) and arsenic to some extent (preys 2 and 6). No biomagnification was observed in the muscles of *S. chuatsi.* Similarly, low biomagnification was observed between the species *C. aumtus* (prey) and *E. ilishaeformis* (predator).

### Total Hazard quotient

Carp (*A. nobilis, C. aumtus,* and *C. carpio*), tilapia (*O. mossambicus*), shrimp and crab muscles were selected to calculate the THQ as they are the most consumed aquatic species in the region. The THQ of arsenic and thallium all exceeded 1, which implied a potential health risk to human beings. Besides, in shrimps and crabs, the THQ values were >1  for cadmium, copper, and zinc, much higher than the other species. In fact, shrimps and crabs had higher THQ values than fish in most cases (arsenic, cadmium, copper, thallium, and zinc). The THQ values for chromium, nickel, and lead were <1 for most of the aquatic organisms implying that adverse health effects were unlikely from the presence of these metals.

### Metal pollution index

**Fig. 4:**
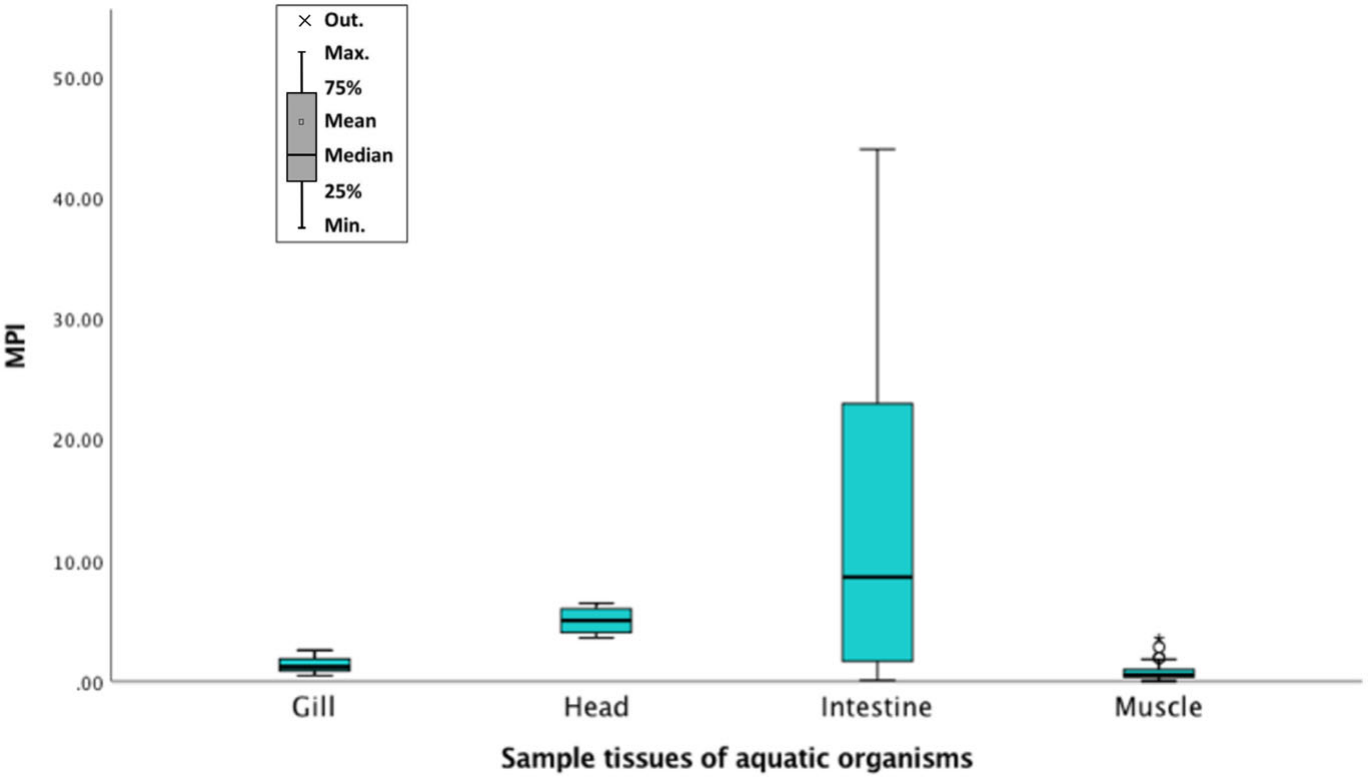
Maximum Pollution Index (MPI) in gills, head, intestine, and muscle samples of aquatic organisms from the Hengshi and Wengjiang Rivers near Dabaoshan mine, China

The highest MPI was found in the intestine of *X. argentea* from site 1, followed by the intestine of snails and *O. mossambicus* from site W*.* High MPI values occurred in all the intestine samples except for shrimp heads from site 4. The MPI values in the species indicated the following order: intestines > gills > muscles (Fig. 4). This trend implied that the accumulation of metals and total metal content were the highest in intestines. Notably, crustaceans and bottom feeders like *P. fulvidraco* and *X. argentea* had high MPI value overall compared to all the other organisms (Tables 3, 4, 5).

**Table 3:**
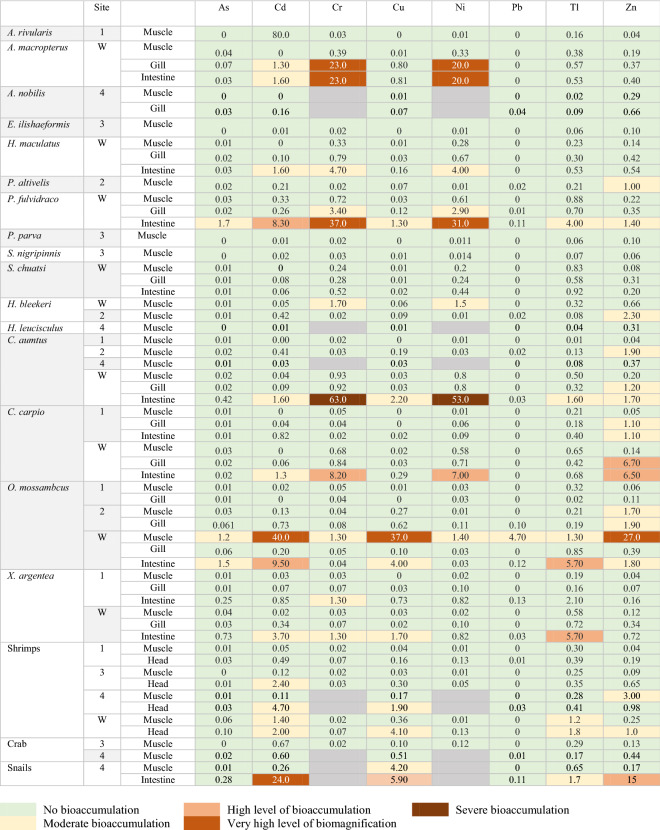
Bioaccumulation factors of metals in aquatic species collected from sites W, 1, 2, 3, and 4 along the Hengshi and Wengjiang Rivers near Dabaoshan mine, China

**Table 4:**
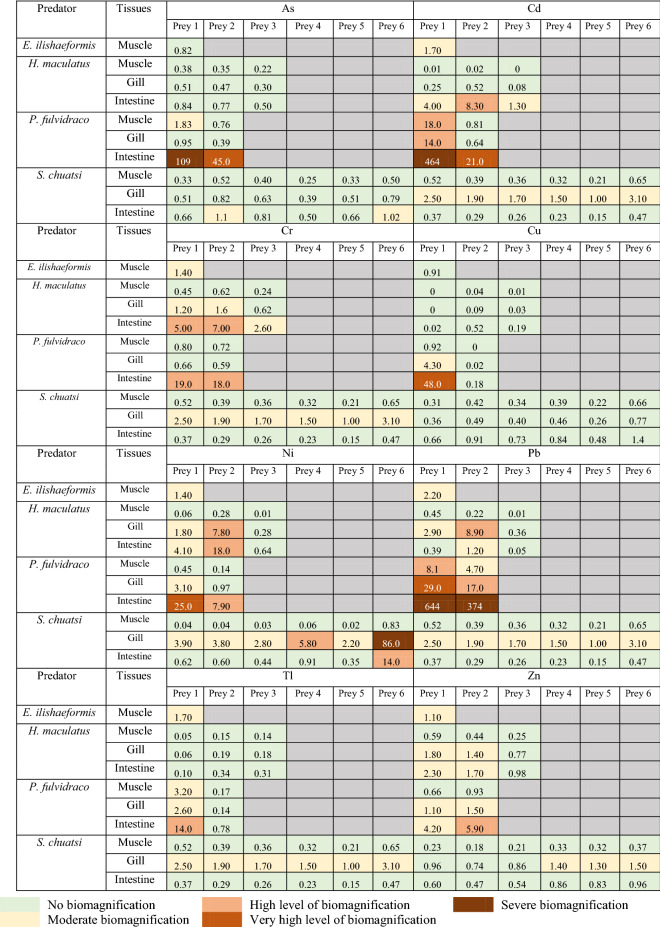
Biomagnification factor of metals in the predatory species collected from the Hengshi and Wengjiang Rivers near Dabaoshan mine, China

**Table 5:**
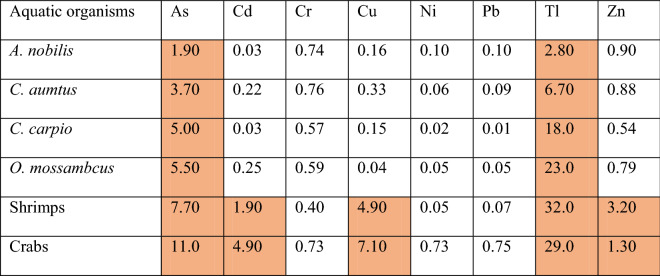
Total Hazard Quotient in the more vulnerable aquatic organisms such as carp, tilapia, shrimp, and crab muscles collected from the Hengshi and Wengjiang Rivers near Dabaoshan mine, China

## Discussion

Despite the fact that the mine has been operational for several decades and extensive metal pollution was reported from this site, there are hardly any systematic studies on AMD effects on aquatic organisms in rivers adjacent to DMS. In this context, the new data on metal contamination in aquatic organisms at DMS provide a holistic insight into monitoring studies applied to other AMD-affected sites in the region. Notably, fishing is one of the critical livelihood resources in the catchment. Local fishermen who helped in sampling indicated that fish caught in the rivers were consumed in the households and sold in the local markets and restaurants. However, it is difficult to infer broad trophic level changes based on the different aquatic species, physical characteristics, and metal concentrations in tissues based on the current data. Nevertheless, field observation and geochemical analyses indicate their status, particularly the safety of these organisms because they are part of the staple diet. Moreover, the trends observed in this study identify the aquatic species that are most vulnerable to metal toxicity, and the dangers posed based on the pollution indices are discussed below.

### Metal concentrations in aquatic organisms

The presence of metals in aquatic species indicates the pathways through which it accumulates in an organism. For example, the study of gills reveals the path of metals uptake through breathing, whereas intestines trace the path of metals uptake, indicating feeding habits and preferences of the organism. The study of different tissues suggests if these parts are safe to be consumed.

The concentration of metals in fish was the highest in the intestines, followed by gills and muscles. In shrimps, higher concentrations of metals occurred in heads instead of muscles. This trend is reflected in Fig. 3, whereby most of the gills and muscles clustered together; shrimp heads belonged to the same group. On the other hand, the fish intestine samples were very different and formed their cluster with two outliers with very high concentrations of metals (Fig. 3). Moreover, MPI calculated in these organisms supported the findings that intestine tissues had the highest total metal concentrations, followed by gills and muscles (Fig. 4 and supplementary data Table S.8). The distribution of metals in the body tissues of aquatic organisms at DMS is consistent with other reports, e.g. Jarić et al. (2011), Zhang et al. (2007), Olaifa et al. (2004). Similarly, a study carried out in the DMS by Zhuang et al. (2013) reported higher metal concentrations in intestines and the lowest level in muscles in aquaculture ponds. The very high concentrations of metals in the intestines could be because the organism has a relatively high potential to accumulate metals when they forage or consume contaminated food (Deb & Fukushima, 1999). The gills also contained high concentrations of metals because of the high adsorption capacity for metals on the surface of gills in contaminated water bodies (Canli & Atli, 2003; Zhang et al., 2007). Besides, gills are also the most exposed organ in the water column to metals.

In general, metal concentrations in the muscles of aquatic organisms are of concern because it is the part that human beings consume the most. The results in this study indicate that several muscle samples do not meet the Chinese and the other international standards, especially for zinc (see supplementary data Tables S1–S4). Despite this, the overall low concentrations of metals in muscles in this study are similar to those reported in previous studies such as Yilmaz (2003) and Zhuang et al. (2013). Muscles tend to accumulate low concentrations of metals because metabolic activities in muscles are generally low (Uluturhan & Kucuksezgin, 2007). Although metal concentrations in muscles are lower than other tissues, caution should be exercised in consuming fish caught from rivers close to AMD-affected sites.

### Metal concentrations in different sites

**Fig. 5:**
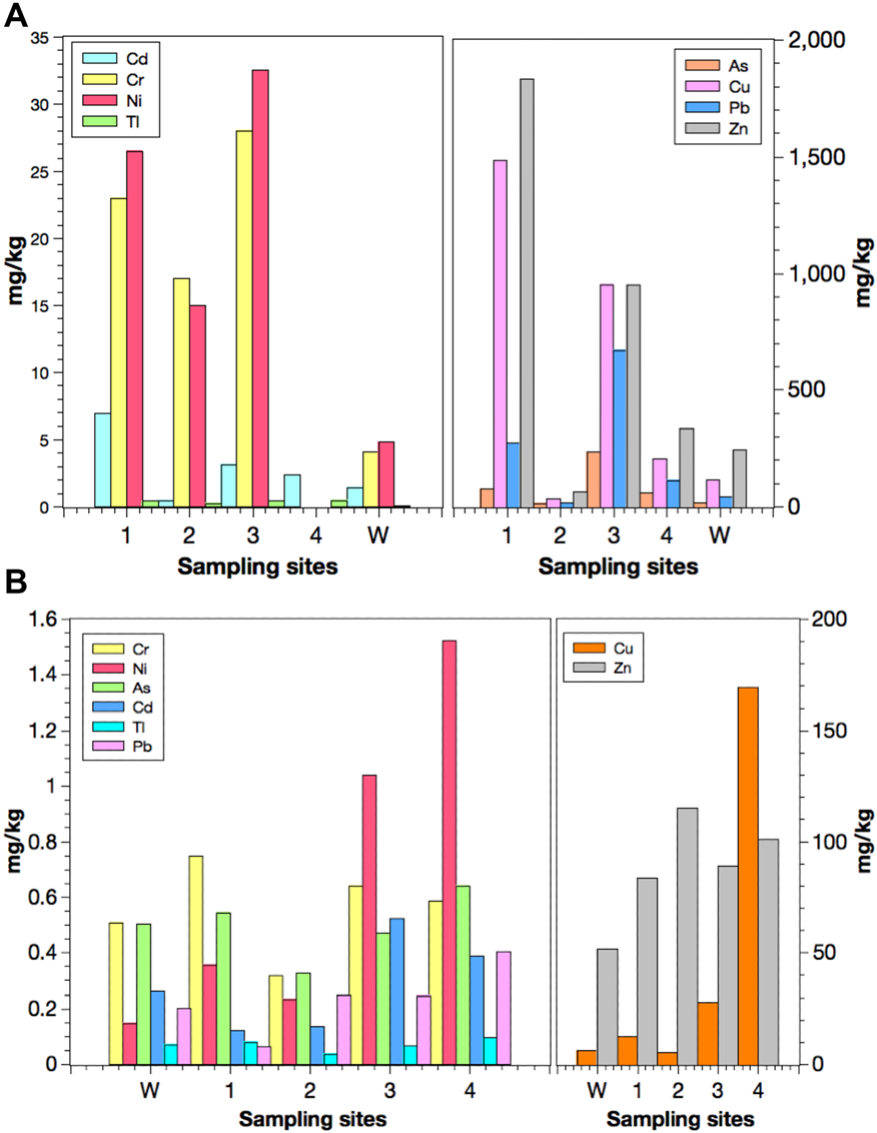
Metal in samples collected from different sites in the Hengshi and Wengjiang Rivers near Dabaoshan mine, China. Metal concentrations (mg/kg) in (**a**) sediments and (**b**) muscles of aquatic organisms

The water samples at sites W and 1 indicated a higher concentration of metals. The sediments at site 3 are the most polluted, whereas sites 2 and W were the least and second least polluted locations, respectively (Fig. 5). When comparing the different sites, it is observed that aquatic organisms collected from site W had the lowest metal concentrations in most cases, except for the muscles in shrimp. Aquatic organisms from site 2 had relatively lower metal concentrations than sites 1, 3, and 4 except zinc (Fig. 5).

Spatial variability observed in the metal trends implies two interesting aspects. First, metal concentrations in sediments and aquatic organisms increase downstream. Second, metals in sediments mostly follow the high concentrations measured in aquatic organisms at the sampling locations. Consistent with this observation, the correlation coefficient between the overall metal concentrations in muscles of aquatic organisms and sediments was 0.49, which is a moderate correlation (Table 6). The low metal concentration in site W is because of the shallow water level (<2 m deep as measured during sampling). It is likely that most contaminants are probably transported downstream by the river as dissolved or bedload fractions. The low concentration in site 2 is because this sample is from the Wengjiang River before its confluence with the contaminated Hengshi River (sites 3 and 4). The lower metal concentrations could be explained by the fact that it was in the upstream section of the Wengjiang River (Fig. 1), and it hardly receives AMD-related discharge from the mine. The primary sources of metal input are household waste from the surrounding villages and agricultural practices in the catchment.

The positive correlation (0.49; Table 6) observed between metals in sediments and aquatic organisms could be explained based on the geochemical properties of sediments. Fluvial sediments are a sink for metals due to their large surface area (silt and clay-rich matrix) that can easily bind them (Gupta et al., 2014). Sediments transport about 30–98% of metals in fluvial systems. When aquatic organisms are feeding at the bottom of the river, metals are ingested together with their food (Gupta et al., 2009, 2014), resulting in high metal concentrations in their body tissues. For example, in this study, a strong positive correlation (0.93; Table 6) occurs between thallium in sediments and muscle samples. The higher the thallium concentration in sediments, the higher it is in aquatic organisms. Consistent with this observation, thallium levels were the highest in site 3 in both sediment and muscle samples. The same reasoning applies to arsenic (*r* = 0.77) and nickel (*r* = 0.75), showing a high correlation. In contrast, the correlation between metals and aquatic organisms is weak for copper and lead, and it is almost non-existent in cadmium. These results imply that metal concentrations and their distribution at DMS are affected by an array of complex physical and biogeochemical processes that are further discussed below.

Even though site W is the closest sampling site to DMS, it has the lowest concentration of metals in water, sediments, and muscles of different aquatic organisms. This trend suggests a strong positive correlation between distance and overall metal concentrations. Based on this, we can infer that a short distance from the mine corresponds to lower metal concentrations in aquatic organisms. The strongest correlation occurs in copper, followed by nickel, lead, thallium, and arsenic. The positive correlation between the distance of the specific site from DMS and metal concentrations in aquatic organisms is interesting. Unlike sediments, aquatic organisms move freely in the river (within their territory). Hence, it is hard to establish a direct relationship between distance from the mine and aquatic organisms and metal concentrations in body tissues. Therefore, metal concentrations in the five sampling sites and the Hengshi and Wengjiang River sediments and the aquatic organisms investigated in this study are inconsistent. Metal concentrations in sediments and aquatic organisms are probably influenced by other factors such as water flow (current), water depth, grain size, organic matter content, temperature, pH, availability of food, and predatory aquatic species at respective sites. A fast stream or base flow in the river is expected to affect the overall amount of metals in sediments. For example, a fast-flowing, more turbulent river will carry metals further away from the point source to other parts of the river (Paronda et al., 2019). At DMS, the inclination of the surface and the strong water currents most likely transport metals away from the mine along the Hengshi and Wengjiang Rivers further downstream in the direction of sampling location site 4. Moreover, metal concentrations in sediments are proposed to increase with a decrease in particle size and an increase in organic matter content (Paronda et al., 2019). Consistent with this idea, site W, which had coarser sediments and low organic matter content compared to the other sites (Luo et al., 2020), resulted in low metal concentrations. Because size and metal content in sediments vary, it will influence the amount of metals ingested by aquatic organisms when they forage.

The differences in weather could influence metal exposure and accumulation in aquatic organisms and sediments. Sampling at site W was performed in winter (early January), while the other sites were sampled in summer (late June). The low metal concentrations in sediments at site W could be explained by the colder and drier weather associated with low streamflow in the Hengshi River. Consistent with this idea, Qin et al. (2019) reported low concentrations of nickel, zinc, lead, and arsenic in samples collected during the dry season with low precipitation resulting in an overall low flowrate and discharge in the stream. As a result, although the low water flow decreases the mobility of metals, it also decreases metals flushed into the river from the mine (as during heavy rains). Consequently, this lowers the general level of metals along the river course. On the other hand, due to heavy rainfall during summer, the base flow increases, and the river becomes more turbulent, which increases currents and discharge of metals that are flushed from the mine site due to enhanced weathering and displacement of tailings into the river (Luo et al., 2020). This results in higher metal concentrations in water and sediment samples collected in June from sites 1 to 4. Moreover, Luo et al. (2020) have suggested that the heavy rainfall during summer in Guangdong province causes an overflow of the treated AMD waste that is discharged into the Hengshi River from the mine. This trend causes an upsurge of metals in the wastewater treatment plant, causing a discharge of insufficiently treated wastewater into the Hengshi River that increased metal concentrations in samples collected in late June. Additionally, the rainy season also reduced the pH in water due to AMD and further enhanced the mobility of metals.

Apart from the difference in precipitation, the contrast in temperature during January and June could cause variation in metal concentrations in sediments. The high temperature during summer increases evaporation which intensifies the amount of metal ions settling from the water column into fluvial sediments and increases the metal concentrations (Paronda et al., 2019). For aquatic organisms, similar observations were reported by Derrag et al. (2014). The authors concluded that metal levels in fish decreased during the winter. Our study showed similar trends as Derrag et al. (2014), whereby average cadmium, copper, zinc, and nickel in aquatic organisms were lower in winter than in summer. Yang and Chen (1996) also reported that high temperature promotes cadmium accumulation in aquatic organisms. It is indicated that there is an increase in the physiological activity (e.g. spawning and foraging) in aquatic organisms during summer because of warmer temperature, which triggers biological changes causing further exposure and a higher uptake of metals (Derrag et al., 2014). In Guangdong province, June to mid-August is the typical spawning season for fish.

The low pH level could increase metals' translocation capacity. This trend would increase solubility and influence bioavailability. The high solubility will result in enhanced metal concentrations in water but comparatively low levels in sediments (Zhuang et al., 2014). Accordingly, the difference in metal concentrations in sediments and the water column will influence metals consumed or adsorbed by different aquatic organisms. For example, a decrease in metal concentrations in sediments will reduce the amount of metal intake by bottom feeders. In contrast, an increase of metals in the water column will increase the adsorption of metals by gills. Hence, organisms with a larger surface area of gills may accumulate more metals. Luo et al. (2020) stated that an increase in metal concentrations in sediments was observed when pH increased; this was due to the co-precipitation of metals under high pH from liming near the mine. Local conditions as weather also had an impact on pH. For example, during heavy rainfall, a large amount of stormwater from the catchment drains into the river, decreasing its pH (Luo et al., 2020; Zhuang et al., 2014).

Finally, remobilization and dispersion of metals are influenced by the type of aquatic organisms and human practices (Karadede-Akin & Ünlü, 2007; Tabinda et al., 2013). Movement of aquatic organisms (e.g. feeding, burrowing) and disturbance caused by human practices such as fishing and transport cause metals loosely bonded to the sediment surface to be released back into the water column. The differences in aquatic organisms collected from different sampling sites and their territorial habitat created a large variability in the average metal concentrations found in muscle tissues. In general, benthic fauna such as mussels, snails, and crustaceans have a higher concentration of metals, followed by demersal fishes (e.g. Crucian carp and catfish). The bottom feeders consume food with high concentrations of metals associated with sediment debris, algae, and insects (Li et al., 2015; Liu et al., 2018; Yi et al., 2011; Zeng et al., 2013). Hence, aquatic species that reside in the bottom waters show higher levels of metals in their tissues than organisms inhabiting the shallow depths of the water column.

### Metals in sediments and organisms

The metal concentrations in sediments indicate that copper, lead, and zinc concentrations were relatively higher in bottom sediments at the polluted sites, suggesting that these metals tend to sink to the bottom and accumulate. For metals in aquatic organisms, it is noted that arsenic, copper, lead, nickel, and thallium levels were higher at site 4, which is farthest away from DMS and is located along the lower reaches of the Wengjiang River. According to Yi et al. (2011), there could be a difference in metal concentrations in different parts of the river, e.g. higher mean concentrations of arsenic occur in the lower reaches of the river. This trend is because of the heterogeneity in physical and chemical properties of sediments, source inputs, biogeochemical processes, and the presence of dominant aquatic organisms at the site.

It was observed that sediments and tissues in aquatic organisms contain high concentrations of arsenic and copper over the safe threshold. In contrast, cadmium is at the medium level, and chromium and nickel are the least severe. Consistent with this trend, previous researchers have indicated higher concentrations of copper, arsenic, and zinc in sediments near the Dabaoshan mine (Luo et al., 2020) and related this to the high concentrations of these metals in aquatic organisms (Zhuang et al., 2013). The high levels of arsenic could be due to the presence of organic arsenicals and microbial activity that releases arsenic in sedimentary environments (Carbonell-Barrachina et al., 2000; Ghosh et al., 2015; Routh & Hjelmquist, 2011). Meador et al. (2004) indicated that arsenic in fish is more related to the dietary uptake of arsenic than the uptake through its gills, which explains the positive correlation coefficient (0.77) between arsenic in sediments and aquatic organisms (Table 6). This also explains why a higher level of arsenic in sediments corresponds to a higher arsenic concentration in aquatic organisms at the same sampling site.

**Table 6:** Correlation coefficients between metal concentrations in muscles of aquatic organisms and metal concentrations in sediments and the distance between the sampling site and Dabaoshan mine in southern China

	Metal concentrations in muscle of aquatic organisms								
	As	Cd	Cr	Cu	Ni	Pb	Tl	Zn	Overall heavy metal concentrations
*Correlation factors*									
Metal concentrations in sediments	0.77	−0.51	0.57	0.08	0.75	0.13	0.93	−0.20	0.49
Distance between sampling site and Dabaoshan mine	0.64	0.32	−0.08	0.97	0.88	0.79	0.66	0.62	0.97

The accumulation of zinc in river sediments is usually not significant (Lewis & McIntosh, 1986). In aquatic organisms, the uptake of zinc generally occurs through gills and gastrointestinal systems, which explains the high zinc levels in the intestines. Adsorption of zinc by gills is also dependent on the chemical composition in water; low calcium concentration in water can critically increase uptake of zinc and its accumulation in gills (Bradley & Sprague, 1985). Although zinc concentration in gills is not high (except for *C. carpio*), a moderate level of zinc bioaccumulates in gills (Table 3). Indeed, a rapid decline of calcium levels observed downstream in the river (Luo et al., 2020) coincides with the increase of zinc in aquatic organisms (lime is added in the upstream section near the mine as part of AMD remediation). This trend supports the connection between declining calcium levels and bioaccumulation of zinc in gills downstream (Bradley & Sprague, 1985).

The high concentration of copper is possibly due to the input of wastewater into the river from mining/ore processing operations at DMS (Luo et al., 2020). Both copper and cadmium can easily bioaccumulate in aquatic organisms because copper is absorbed by aquatic species and transported by blood plasma. In contrast, cadmium has a high affinity for internal organs to which they can readily adsorb (Kondera et al., 2014). This explanation supports the high bioaccumulation of cadmium and copper in aquatic organisms near DMS.

The presence of chromium and nickel shows less acute contamination in water, sediments, and aquatic organisms at the sampling sites. Nonetheless, both metals (Table 3) observed a high bioaccumulation level, and they surpass the permissible safe limits. It was reported that copper increases the uptake and accumulation of nickel in aquatic organisms and algae (Hutchinson & Czyrska, 1975). Thus, the bioaccumulation of nickel in these samples (Table 3) could be explained by the high concentration of copper in the environment (Table 2) as well as in the tissues of aquatic organisms. For chromium, the relatively low concentration in the tissues may be caused by the rapid excretion of chromium from blood, gills, and the digestive tract in fish (Van der Putte et al., 1981). Nonetheless, bioaccumulation of chromium in aquatic organisms (Table 3) and biomagnification in the intestines (Table 4) suggest that chromium still poses a safety risk.

In general, the amount of lead in sediments increases with a temperature rise, and a pH value of 4–6 enhances the adsorption capacity of lead (Zhang et al., 2012). Although the concentration of lead in sediments is more severe than that in aquatic organisms, many aquatic organisms do not meet the safe limit for lead. The concentration of lead in sediments is relatively low during winter than in summer. The uptake of lead in aquatic organisms from sediments is usually high at pH 4.5–5.5 (Lewis & McIntosh, 1986). The authors indicated that at pH 5.5, the uptake of lead from sediments is faster than from the water because the lead-iron oxide association is weak under low pH. Hence, the bioavailability of lead increases. Consistent with this trend, the concentration of lead is higher during summer when the pH level is low (Table 1 and Luo et al., 2020).

Thallium shows a robust correlation between metal concentrations in sediments and aquatic species (Table 6). This is because uptake of thallium from sediments to aquatic organisms is very efficient during the feeding process (Lapointe & Couture, 2010). Besides, the input of thallium in sediments and aquatic organisms is most likely from pollution caused by mining or processing ores at DMS. Zhang et al. (1998) suggested that thallium is transported by stream water adjacent to the site due to ongoing mining operations. Notably, in aquatic organisms, the prey can be a fundamental source of thallium; it was further reported that the transfer of thallium from the prey to predator is a highly efficient process (Lapointe & Couture, 2009, 2010). However, the BMF of thallium (Table 4) was not very high compared to other elements. The concentration of thallium in the muscles of some aquatic organisms tends to increase when the dissolved thallium level in water is high. In contrast, the accumulation of thallium in gills is reported to be constant despite the change of thallium levels in water (Zitko et al., 1975). This is also evident in the current study. While there is a slight difference in thallium levels in gills in samples collected during winter and summer, there is a larger difference in muscles.

### Metal concentrations in organisms and their size

Some relationship between the size of organisms and their corresponding metal levels is evident in some aquatic organisms. The difference is most evident in the case of crabs. Crabs from site 4 (which are twice as large) have much higher metal concentrations than the smaller crabs from site 3 (supplementary data Table S.6). Likewise, a matching pattern was observed in thallium and lead concentrations in the tissues of *C. aumtus*. Similar findings were reported by Kwok et al. (2014) that larger fish species contained higher levels of cadmium, copper, chromium, and zinc. In contrast, *X. argentea* from site 1 indicated higher concentrations of all metals except arsenic in its muscles than site W, even though *X. argentea* from site W is larger (supplementary data Table S.6). Therefore, a negative correlation exists between the size of *X. argentea* and metals in its tissue. A similar observation is noted in the tissues of *C. carpio* except for copper, arsenic, and lead in its muscles. It is suggested that several aquatic organisms have a positive correlation between their size and the concentration of metals in their tissues. However, in a few species, low metal concentrations are prevalent in larger individuals. Newman and Unger (2003) have stated that high concentrations of metals reported in larger individuals are due to their long exposure to a polluted environment. Moreover, the large surface area in bigger individuals leads to higher adsorption of metals. The size of larger organs, tissues like muscles, skin, and fat facilitate more metals to be stored in the organism’s body. For some aquatic organisms, the negative correlation between their size and metals that have accumulated in their body is explained by the dilution effect it undergoes from rapid increase of size in the organism, especially in many fast-growing species such as catfish and tilapia (Jardine et al., 2009; Newman & Unger, 2003).

### Metal concentrations in organisms and their habitats

The results indicate that the intestines of *X. argentea* from site 1 and *O. mossambicus* from site 2 contained high concentrations of metals such as chromium, copper, nickel, and thallium in *X. argentea,* and copper, arsenic, and lead in *O. mossambicus*. In addition, the intestines in snails indicated the highest levels of zinc and cadmium (supplementary data Table S.1). The overall levels of metals were the highest in the intestines of snails, followed by *X. argentea, O. mossambicus,* and *P. fulvidraco*. Notably, the high concentrations of metals in the intestines were higher than all other tissues. Likewise, the MPI values also suggested that crustaceans and bottom feeders like *P. fulvidraco* and *X. argentea* have a higher level of total metal content (supplementary data Table S.8).

*X. argentea* is an omnivorous bottom-feeding fish (supplementary data Table S.6). Its diet consists of periphyton algae and organic debris (Peng et al., 2018). Hence, the bottom feeders generally have an elevated level of metal accumulation in their bodies (Li et al., 2015; Liu et al., 2018; Yi et al., 2011; Zeng et al., 2013). This is reflected by the high concentrations of metals present in the intestines of *X. argentea.* Moreover, *X. argentea* is a relatively big fish (supplementary data Table S.6), and thus, more metals accumulate with age in its tissues. Similarly, *O. mossambicus* is an omnivorous fish that feeds on algae, debris, and zooplankton in the middle-lower part of the water column (supplementary data Table S.6), which explains the high concentrations of metals found in its intestines. Moreover, *O. mossambicus* caught in site 2 is relatively large compared to those collected from the other sites, indicating the possibility that its large size partly contributed to the high amount of metals found in its body.

**Fig. 6:**
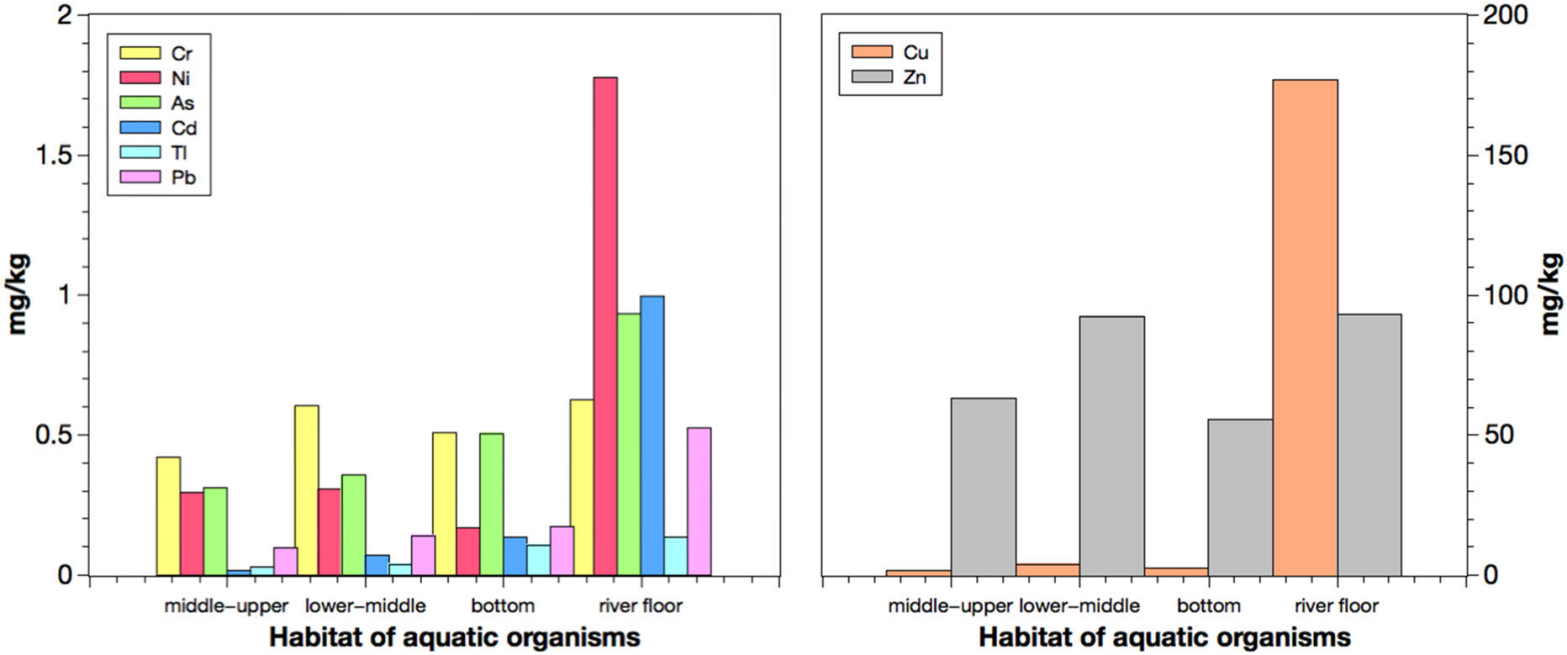
Metal concentrations (mg/kg) in aquatic organisms from different habitats in the Hengshi and Wengjiang Rivers near Dabaoshan mine, China

The metal levels in gills were compared, and it was noted that *A. nobilis* (site 4) shows the highest concentrations of chromium, nickel, and lead. In contrast, *A. macropterus* (site W) has the most elevated copper, arsenic, and cadmium levels. Both are omnivores that feed in the middle and upper parts of the water column. The high concentration of metals in its gills indicates a high metal uptake from surface waters. A common factor leading to an increase in metals in surface water could be the low pH favouring the release of metals from sediments. In general, the low pH level could be caused by the wastewater released from the mine or its migration during spells of heavy rainfall in the province (Luo et al., 2020) (Fig. 6).

Notably, crabs and shrimps indicate the highest concentrations of most metals except for chromium, thallium, and zinc. High levels of these metals also occur in *C. aumtus, P. fulvidraco,* and *H. bleekeri*, which are all bottom feeders except *H. bleekeri* (supplementary data Table S.6). Because the intestines of shrimps and crabs collected were too small, only muscle samples and shrimp heads were analysed. Besides, many samples had to be caught to get enough soft tissue for freeze-drying and metal analyses, which was ethically unacceptable. Moreover, it has been reported in many studies that higher metal concentrations in muscles imply higher concentrations in its intestines (Jarić et al., 2011; Olaifa et al., 2004; Zhang et al., 2007). The benthic fauna, including crabs and shrimps living on the riverbed, are exposed to contaminant-rich sediments, particularly when food is ingested, resulting in the accumulation of metals in their tissues (Li et al., 2015; Liu et al., 2018). It is also reported that demersal fish and benthic fauna generally have higher concentrations of arsenic, cadmium, chromium, copper, and zinc (Yi et al., 2011), which matched the results in this study.

It is noted that *S. chuatsi* contains the lowest metal concentrations in most cases. According to Liang et al. (1998), *S. chuatsi* has a special feeding habit as they only feed on fry (juvenile fish), which typically do not contain high metal concentrations. Because *S. chuatsi* feeds on prey with low contamination levels and resides in the middle-upper part of the water column (supplementary data Table S.6), the exposure to dissolved metals decreases. In contrast, carnivores and predatory fish have a higher chance of accumulating more metals in their bodies because of the higher trophic level as part of the biomagnification effect in the food chain (Martin & Griswold, 2009). This trend is not readily evident in this study. The feeding habit and diet likely affect the metal accumulation and its biomagnification in the food chain. For example, carnivores like *P. fulvidraco* that feed on juvenile and adult fishes and inhabit the bottom level of the river contain much higher metal concentrations than *S. chuatsi* that feeds on flies and resides in the middle-upper part of the water column.

### Bioaccumulation and biomagnification in aquatic organisms

In general, the results indicate that different organisms at a higher trophic level and feeding the lower-bottom layer of the riverbed tend to show a high value of BAF and BMF. Although site W tends to have a low value of metal concentrations, higher BAFs occur at this site, especially for chromium and nickel (Table 3). Moreover, between the different samples, gills and intestines indicate higher BAFs because of higher metabolic activity in these tissues and their exposure (Table 3).

The aquatic organisms with the highest levels of metal bioaccumulation, i.e. chromium and nickel, are in *P. fulvidraco* (Yellowhead catfish) and *C. aumtus* (Crucian carp) (Table 3). Likewise, *O. mossambicus* indicated a high level of bioaccumulation for copper, cadmium, and zinc. According to Yi et al. (2011), the accumulation of metals in aquatic organisms results from breathing, surface contact with polluted water, and predation. *P. fulvidraco* inhabits bottom sediments (supplementary data Table S.6) and belongs to the upper part of the food chain in an aquatic ecosystem. As a carnivore, *P. fulvidraco* generally feeds on adult fishes. On the other hand, *C. aumtus* is an omnivorous bottom feeder and is one of the prey of *P. fulvidraco*. This observation is consistent with previous studies, whereby carnivores and bottom feeders are reported to have the highest level of bioaccumulation as they consume and accumulate a large number of metals from their prey or sediments ingested during feeding (Li et al., 2015; Liu et al., 2018; Martin & Griswold, 2009).

*S. chuatsi* shows no bioaccumulation sign (Table 3) and has limited biomagnification effect (Table 4) because it undergoes less exposure to metals. When the organisms are at a higher trophic level, they may indicate low bioaccumulation of certain metals. For example, the result showed a high BAF for *H. bleekeri* (a herbivore) than *P. parva* (an omnivore) for arsenic, cadmium, chromium, copper, nickel, and thallium (Table 3). According to Chen and Folt (2000), some metals are not rare to be bio-diminutive in the food web because organisms at a lower trophic level have a larger metal burden than those at the higher trophic level. This is because the lower trophic organisms feed directly from highly contaminated sediments resulting in greater exposure and a higher tendency to accumulate metals in their tissues. Finally, not all the metals in a prey will be transferred to the predator (Chen & Folt, 2000; Hutchinson & Czyrska, 1975). The ease of transferring metals from one trophic level to the next one is dependent on its assimilation capacity. Hence, metals like lead and cadmium have a higher ability to retain their concentration in the food chain compared to other metals (Couture et al., 2011).

### Safety risk

**Fig. 7:**
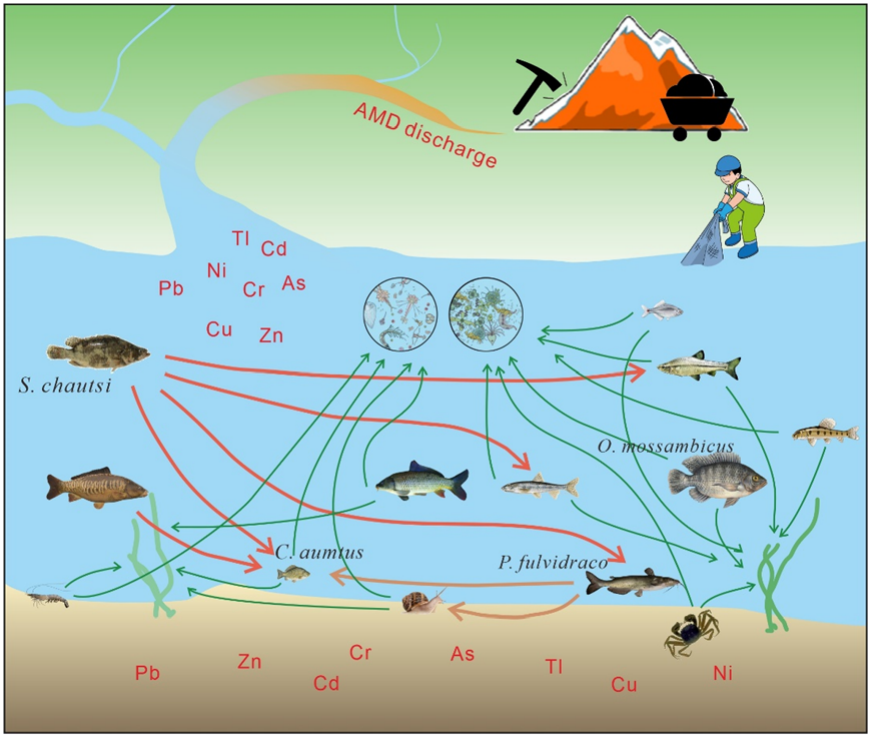
Metal pollution, habitats of aquatic organisms, and predator–prey relationship in the Hengshi and Wengjiang Rivers near Dabaoshan mine, China

Metal concentrations in aquatic organisms are a concern for the public, especially in China, because local people regularly consume fish, shrimps, snails, and crabs consume fish, shrimps, snails, and crabs as part of their routine dietary intake. According to the guidelines mentioned above, most muscle samples are under the permissible safe limits for metal concentrations. However, the results presented in this study suggest that muscles of crustaceans and bottom feeders could still be risky for human consumption (e.g. for arsenic, cadmium, lead, and thallium) and pose a threat to human health due to bioaccumulation and biomagnification effects (Fig. 7). The metal concentrations in gills are of less concern as they are seldom consumed. On the other hand, although fish intestines and shrimp heads are not the most popular parts to be eaten internationally, it is not rare in China to see restaurants offering meals comprised of fish intestines and local people consuming shrimp heads. This is an alarming practice since metals accumulate in intestines as well as in shrimp heads. Consistent with this, none of the intestine samples from aquatic organisms investigated in this study meet the safety standards, and therefore, it is crucial to inform the residents about the potential health threats from including intestines in traditional cuisines.

It is known that carps and tilapias are two of the most common freshwater species consumed by local people (Chiu et al., 2013; Yuan et al., 2001). Both species are bottom or middle-lower level feeders, and they both belong to the middle-upper level position in the food chain. There are chances of high uptake and bioaccumulation of metals in their bodies, which pose a significant risk. It is to be noted that catfish (*P. fulvidraco*), which indicated the highest level of bioaccumulation and its high trophic position in the food chain, is seldom eaten by the local people. Nonetheless, our results show that consuming aquatic species from near the AMD-affected areas near the Dabaoshan mine can be harmful to human beings. In particular, the THQ values calculated in this study suggest that all commonly consumed aquatic species like carps, tilapias, shrimps, and crabs pose a potential health risk, especially the crustaceans (Table 5). Thus, inhabitants should reduce the consumption of local fish and crustaceans owing to the potent levels of metals, particularly arsenic and thallium.

## Conclusions

The Dabaoshan mine affected by AMD release metals in surface water and sediments, which may last for a long time if not properly managed. The abundance of trace metals in the environment indicated the general trend Zn > Cu > Pb > As > Cr > Ni > Cd > Tl. The metal concentrations in sediments and aquatic organisms retrieved from the Hengshi River and Wengjiang River near DMS were high compared to the official standards, particularly for arsenic, copper, and zinc. A strong positive correlation was found between the distance of the sampling sites from DMS and the overall metal concentrations in the muscles of aquatic organisms, particularly for copper and nickel. This trend was affected by numerous factors such as water flow, weather, the size of organisms, and the chemical composition of sediments. There are variations in metal concentrations between the winter and summer seasons; sediments collected during summer had higher concentrations of metals due to the differences in pH, temperature, and precipitation that affect the distribution of metals.

A positive correlation occurred between metal concentrations in sediments and aquatic organisms, particularly thallium, arsenic, and nickel. Several aquatic species indicated a positive correlation between their size and concentrations of certain metals in their tissues, mainly due to the long exposure time in the polluted AMD waters in larger species. The intestines in aquatic organisms contained the highest concentrations of most metals analysed in this study. Besides, gills and heads of shrimps indicated high concentrations of metals. However, gills and heads are of less concern because they are seldom consumed. On the other hand, in several cases of fish, the muscle tissues exceeded the safety limits, especially for zinc and copper. This trend is of concern because local people consume these organisms as a rich source of proteins. Although most muscle samples met the recommended limits for dietary intake, intestines, heads, and gills are occasionally consumed and pose a risk if human beings eat them.

The results confirmed that feeding habits and the habitat of aquatic organisms greatly influenced metal concentrations and the bioaccumulation process. Thus, demersal fish and benthic fauna generally had higher concentrations of metals in their tissues. In contrast, *S. chuatsi,* which feeds on juvenile fish and resides in the middle-upper part of the water column, had a low concentration of most metals in its tissues. Bottom feeders like *C. aumtus* and *X. argentea* indicated higher bioaccumulation levels (Cr > Ni > Cd > Cu > Zn > Tl > Pb > As). In addition, organisms from the upper trophic level like *P. fulvidraco* and *O. mossambicus* indicated high biomagnification levels. Lead and cadmium indicated the highest level of biomagnification from prey to predator. Overall, metal accumulation in aquatic organisms in the study area because of mining operations poses an ecological issue and potential health risks arising from contamination of arsenic, cadmium, lead, thallium, and other metals.

## Supplementary Information

Below is the link to the electronic supplementary material.

Supplementary file1 (DOCX 4036 kb)
