# Supplementary material for: Metal accumulations in aquatic organisms and health risks in an acid mine-affected site in South China
Source: Environ Geochem Health. 2021 Apr 19;43(11):4415–40. doi: 10.1007/s10653-021-00923-0 (PMC8528778; doi:10.1007/s10653-021-00923-0)
Supplement: Supplementary file 1 — Supplementary file1 (DOCX 4036 kb) [file 10653_2021_923_MOESM1_ESM.docx]

**Supplementary Data**

| Site | Aquatic organisms Tissue | | Cr | Ni | Cu | Zn | As | Cd | Tl | Pb |  |
| --- | --- | --- | --- | --- | --- | --- | --- | --- | --- | --- | --- |
| W | *Carassius aumtus* | muscle | 0.17-1.90 | 0.01-0.07 | 3.05-3.80 | 48.0 | 0.24-0.32 | 0.01-0.05 | 0.023-0.030 | BDL-0.09 |  |
|  |  | gill | 0.63 | 0.44 | 3.80 | 283 | 0.46 | 0.13 | 0.02 | 0.94 |  |
|  |  | intestine | 4.90 | 2.40 | 258 | 424 | 8.00 | 2.30 | 0.10 | 15.0 |  |
|  | *Oreochroms mossambcus* | muscle | 0.29 | 0.12 | 4.1 | 27.0 | 0.59 | 0.08 | 0.07 | 0.02 |  |
|  |  | gill | 0.89 | 1.01 | 11.0 | 96.0 | 1.10 | 0.28 | 0.05 | 1.40 |  |
|  |  | intestine | 16.0 | 13.0 | 470 | 446 | 28.0 | 13.0 | 0.34 | 53.0 |  |
|  | *Pseudobagrus fulvidraco* | muscle | 0.30-0.38 | 0.01-0.12 | 1.50-2.90 | 51.0-54.0 | 0.19-0.54 | 0.01-0.46 | 0.04-0.05 | BDL-0.66 |  |
|  |  | gill | 0.24-0.61 | 0.37-0.80 | 2.80-13.0 | 61.0-86.0 | 0.18-0.28 | 0.07-0.37 | 0.03-0.04 | 5.70-0.36 |  |
|  |  | intestine | 0.60-7.2 | 0.67-6.50 | 14.0-152 | 151-342 | 0.59-32 | 0.56-12.0 | 0.09-0.24 | 0.30-52.0 |  |
|  | *Xenocypris argentea* | muscle | 0.43 | 0.08 | 3.10 | 31.0 | 0.68 | 0.03 | 0.04 | BDL |  |
|  |  | gill | 0.61 | 1.10 | 2.50 | 84.0 | 0.66 | 0.49 | 0.04 | 0.32 |  |
|  |  | intestine | 29.0 | 42.0 | 196 | 178 | 14.0 | 5.30 | 0.34 | 15.0 |  |
|  | *Siniperca chuatsi* | muscle | 0.19 | BDL | 0.99 | 19 | 0.10 | BDL | 0.05 | BDL |  |
|  |  | gill | 0.92 | 1.00 | 1.2 | 77 | 0.15 | 0.11 | 0.04 | 0.06 |  |
|  |  | intestine | 0.14 | 0.16 | 2.1 | 49 | 0.2 | 0.08 | 0.06 | BDL |  |
|  | *Acheilognathus macropterus* | muscle | 0.16 | 0.10 | 1.6 | 47 | 0.7 | 0 | 0.02 | 0.05 |  |
|  |  | gill | 0.5 | 0.94 | 95.0 | 91 | 1.4 | 1.83 | 0.03 | 0.52 |  |
|  |  | intestine | 2.1 | 1.10 | 96.0 | 98 | 0.6 | 2.30 | 0.03 | 0.05 |  |
|  | *Hemibarbus maculatus* | muscle | 0.19 | 0.05 | 1.30 | 34 | 0.27 | 0 | 0.01 | BDL |  |
|  |  | gill | 0.49 | 1.50 | 3.20 | 104 | 0.36 | 0.14 | 0.02 | 0.40 |  |
|  |  | intestine | 2.10 | 3.40 | 19.0 | 133 | 0.6 | 2.3 | 0.03 | 0.05 |  |
|  | Shrimps | muscle | 0.18-1.00 | 0.12-0.44 | 34.0 -42.0 | 56-62 | 0.92-1.2 | 0.21-1.9 | 0.06-0.07 | BDL-1.06 |  |
|  |  | head | 0.70-2.20 | 2.50-2.90 | 468-480 | 207 -255 | 1.9-1.9 | 1.9-2.9 | 0.09-0.11 | 0.01-0.06 |  |
|  | *Hemiculter bleekeri* | muscle | 0.33 | 0.48 | 7.10 | 161 | 0.21 | 0.07 | 0.02 | 0.07 |  |
|  | *Cyprinus carpio* | muscle | 0.20 | BDL | 2.80 | 35.0 | 0.49 | 0.01 | 0.04 | BDL |  |
|  |  | gill | 1.00 | 1.30 | 3.50 | 1655 | 0.41 | 0.08 | 0.03 | 0.58 |  |
|  |  | intestine | 0.37 | 1.30 | 34.0 | 1599 | 0.31 | 1.80 | 0.04 | 0.14 |  |
| Measured values for certified reference materials (certified values in parenthesis) | | | | | | | | | | | |
| GBW 08573 Standard | | | 0.19  (0.43) | 1.06  (1.50) | 1.79  (1.36) | 27.3  (28.8) | 6.6  (5.08) | 0.007  (0.015) | 0.001  (-) | BDL  (-) |  |

Table S.1. Heavy metal concentrations (mg/kg dry weight) in different tissues of aquatic species collected from site W in the Hengshi and Wengjiang Rivers near Dabaoshan mine, Guangdong province, China. BDL – below the detection limit. The standard reference material (GBW 08573) was used for QA/QC.

| Site | Aquatic organisms Tissue | | Cr | Ni | Cu | Zn | As | Cd | Tl | Pb |
| --- | --- | --- | --- | --- | --- | --- | --- | --- | --- | --- |
| 1 | *Cyprinus carpio* | muscle | 1.02 | 0.23 | 1.40 | 82.0 | 0.58 | 0.01 | 0.09 | 0.04 |
|  |  | gill | 0.90 | 1.50 | 2.90 | 1960 | 0.39 | 0.28 | 0.07 | 0.56 |
|  |  | intestine | 0.45 | 2.40 | 34.0 | 2027 | 0.59 | 5.67 | 0.16 | 0.18 |
|  | *Xenocypris argentea* | muscle | 0.51-0.75 | 0.41-0.46 | 3.20-4.90 | 48.0-63.0 | 0.42-0.42 | 0.12-0.19 | 0.03-0.08 | 0.04-0.08 |
|  |  | gill | 1.40-1.70 | 1.90-2.60 | 4.00-43.0 | 117-120 | 0.53-1.10 | 0.26-0.49 | 0.05-0.06 | 0.54-1.20 |
|  |  | intestine | 0.45-29.0 | 2.10-22.0 | 110-1075 | 125-283 | 0.25-420 | 0.94-5.90 | 0.12-0.83 | 2.90-35.0 |
|  | *Oreochroms mossambcus* | muscle | 0.72-1.10 | 0.36-0.77 | 6.10-10.0 | 68.0-117 | 0.48-0.69 | 0.06-0.13 | 0.06-0.13 | 0.06-0.14 |
|  |  | gill | 0.89 | 0.84 | 3.10 | 198 | 0.45 | 0.02 | 0.01 | 0.82 |
|  | *Abbottina rivularis* | muscle | 0.75 | 0.21 | 3.90 | 80.0 | 0.34 | 0.05 | 0.06 | 0.03 |
|  | *Carassius aumtus* | muscle | 0.41 | 0.32 | 2.20 | 80.0 | 0.39 | BDL | 0.00 | 0.02 |
|  | Shrimps | muscle | 0.48 | 0.23 | 55.0 | 78.0 | 0.84 | 0.35 | 0.12 | 0.11 |
|  |  | head | 1.70 | 3.40 | 243 | 347 | 2.00 | 3.40 | 0.16 | 1.70 |

Table S.2. Heavy metal concentrations (mg/kg dry weight) in different tissues of aquatic species collected from site 1 in the Hengshi and Wengjiang Rivers near Dabaoshan mine, Guangdong province, China. BDL – below the detection limit. Standard reference material (GBW 08573) was used.

| Site | Aquatic organisms Tissue | | Cr | Ni | Cu | Zn | As | Cd | Tl | Pb |
| --- | --- | --- | --- | --- | --- | --- | --- | --- | --- | --- |
| 2 | *Oreochroms mossambcus* | muscle | 0.50 | 0.18 | 9.90 | 111 | 0.50 | 0.06 | 0.05 | 0.06 |
|  |  | gill | 0.90 | 1.7 | 23.0 | 127 | 1.00 | 0.34 | 0.05 | 2.00 |
|  |  | intestine | 15.0 | 21.0 | 1346 | 1776 | 21.0 | 18.0 | 0 | 93.0 |
|  | *Carassius aumtus* | muscle | 0.23-0.39 | 0.29-0.44 | 4.30-7.00 | 8.06-126 | 0.28-0.34 | 0.03-0.19 | 0.01-0.03 | 0.34-0.05 |
|  | *Hemiculter bleekeri* | muscle | 0.26 | 0.21 | 3.15 | 153 | 0.15 | 0.19 | 0.02 | 0.30 |
|  | *Plecoglossus altivelis* | muscle | 0.28 | 0.10 | 2.40 | 69.0 | 0.32 | 0.10 | 0.05 | 0.29 |
|  | Shrimps | muscle | 0.32 | 0.64 | 161 | 203 | 1.10 | 1.70 | 0.10 | 0.10 |
| 3 | *Sarcocheilichthys nigripinnis* | muscle | 0.91 | 0.46 | 4.40 | 59.0 | 0.30 | 0.06 | 0.03 | 0.25 |
|  | *Erythroculter ilishaeformis, Pseudorasbora parva* | muscle | 0.53 | 0.37 | 2.90 | 90.0 | 0.24 | 0.04 | 0.03 | 0.18 |
|  | *Crab* | muscle | 0.68 | 3.8 | 99 | 123 | 0.88 | 2.10 | 0.13 | 0.56 |
|  | Shrimps | muscle | 0.24-0.55 | 0.19-0.20 | 28.0-31.0 | 80.0-82.0 | 0.66-0.68 | 0.29-0.37 | 0.10-0.11 | 0.01-0.06 |
|  |  | head | 0.76-0.78 | 1.23-1.70 | 252-286 | 225-620 | 1.20-1.50 | 7.60-7.70 | 0.14-0.15 | 0.36-0.42 |

Table S.3. Heavy metal concentrations (mg/kg dry weight) in different tissues of aquatic species collected from site 2 and site 3 in the Hengshi and Wengjiang Rivers near Dabaoshan mine, Guangdong province, China. Standard reference material (GBW 08573) was used.

| Site | Aquatic organisms Tissue | | Cr | Ni | Cu | Zn | As | Cd | Tl | Pb |
| --- | --- | --- | --- | --- | --- | --- | --- | --- | --- | --- |
| 4 | *Aristichthys nobilis* | muscle | 0.80 | 0.70 | 2.23 | 97.0 | 0.20 | 0.01 | 0.01 | 0.14 |
|  |  | gill | 1.90 | 2.90 | 15.0 | 220 | 1.70 | 0.37 | 0.04 | 4.10 |
|  | *Hemiculter leucisculus* | muscle | 0.49 | 0.27 | 2.30 | 104 | 0.18 | 0.03 | 0.02 | 0.09 |
|  | *Carassius aumtus* | muscle | 0.52-0.55 | 0.42-0.91 | 2.70-6.00 | 100-123 | 0.33-0.55 | 0.04-0.07 | 0.02-0.04 | 0.12-0.35 |
|  | Snails | muscle | 0.41 | 0.83 | 868 | 58.0 | 0.71 | 0.60 | 0.30 | 0.10 |
|  |  | intestine | 7.00 | 17.0 | 1217 | 4981 | 18.0 | 57.0 | 0.78 | 13.0 |
|  | Shrimps | muscle | 0.28-0.37 | 0.20-0.22 | 31.0-34.0 | 92.0-1000 | 0.67-0.70 | 0.25-0.26 | 0.09-0.13 | 0.05-0.10 |
|  |  | head | 0.85-1.20 | 1.50-1.60 | 365-400 | 321-326 | 1.70-1.80 | 11.0 | 0.11-0.19 | 0.96-3.30 |
|  | Crab | muscle | 0.89 | 6.70 | 105 | 147 | 1.50 | 1.40 | 0.078 | 1.60 |

Table S.4. Heavy metal concentrations (mg/kg dry weight) in different tissues of aquatic species collected from site 4 in the Hengshi and Wengjiang Rivers near Dabaoshan mine, Guangdong province, China. Standard reference material (GBW 08573) was used.

| Standards | **As** | **Cd** | **Cr** | **Cu** | **Ni** | **Pb** | **Tl** | **Zn** | **Reference** |
| --- | --- | --- | --- | --- | --- | --- | --- | --- | --- |
| Chinese Standard | 2.50 | 0.50 | 1.00 | 50.0 | 5.00 | 2.50 | - | 100 | (Bugang and Woolsey, 2012, China National Standards Management Department, 2001) |
| UNEP | - | 1.50 | - | - | - | 1.50 | - | - | (UNEP, 1985) |
| IAEA-407 | - | 0.90 | 3.65 | 16.4 | 3.00 | 0.60 | - | - | (Wyse et al., 2003) |
| EC | - | 0.25 | - | 100 | - | 1.00 | - | - | (European Commission, 2005) |
| FAO/WHO | - | 0.25 | - | 150 | - | 2.50 | - | 200 | (Joint FAO/WHO Expert Committee on Food Additives, 2010) |
| EPA | - | - | - | - | - | - | 0.25 | - | (EPA, 2009) |

Table S.5. International standards for heavy metal concentrations (mg/kg dry weight) in aquatic organisms (the conversion factor 5 is used to convert metal concentrations in wet weight to dry weight).

| **Species Name** | **Common Name** | **Feeding Habit** | **Habitat** | **Sites where found** | **Weight (g)** | **Length (cm)** | **Width (cm)** |
| --- | --- | --- | --- | --- | --- | --- | --- |
| *Abbottina rivularis* | Chinese False Gudgeon | Omnivorous | Middle-lower | 1 | 53 | 17 | 3.5 |
| *Acheilognathus macropterus* | Giant Chinese bitterling | Omnivorous | Middle-upper | W | N/A | 9.0 | 3.0-3.5 |
| *Aristichthys nobilis* | Bighead Carp | Omnivorous | Middle-upper | 4 | 55 | 20 | 5.0 |
| *Carassius aumtus* | Crucian Carp | Omnivorous | Bottom | W | N/A | 11-12 | 4.0 |
|  |  |  |  | 1 | 64 | 15 | 5.0 |
|  |  |  |  | 2 | 5.0-31 | 7.0-13 | 2.0-4.0 |
|  |  |  |  | 4 | 6.0 | 7.5 | 2.5 |
| *Cyprinus carpio* | Common Carp | Omnivorous | Middle-lower | W | N/A | 32 | 10 |
|  |  |  |  | 1 | 391 | 29 | 10 |
| *Erythroculter ilishaeformis* | Predatory Carp | Carnivorous | Middle-lower | 3 | 3.0-19 | 3-14 | 1.5-8.0 |
| *Hemibarbus maculatus* | Spotted Steed | Carnivorous | Middle-lower | W | N/A | 25-26 | 4.1-4.5 |
| *Hemiculter bleekeri* | Minnow | Herbivorous | Middle-lower | W | N/A | 8.0-8.5 | 1.5 |
|  |  |  |  | 2 | 6.0 | 9.0 | 2.0 |
| *Hemiculter leucisculus* | Sharpbelly | Omnivorous | Middle-lower | 4 | 13 | 12 | 2.5 |
| *Oreochromis mossambicus* | Mozambique Tilapia | Omnivorous | Middle-lower | W | N/A | 16-17 | 5.5 |
|  |  |  |  | 1 | 18-33 | 11.5-16.5 | 3.0-4.0 |
|  |  |  |  | 2 | 147 | 22 | 7.0 |
| *Plecoglossus altivelis* | Ayu | Omnivorous | Bottom | 2 | 30 | 15 | 5.0 |
| *Pseudobagrus fulvidraco* | Yellowhead catfish | Carnivorous | Bottom | W | N/A | 14-15 | 2.0-2.5 |
| *Pseudorasbora parva* | Stone Moroko | Carnivorous | Middle-upper | 3 | 3.0-19 | 3.0-14 | 1.5-8.0 |
| *Sarcocheilichthys nigripinnis* | Rainbow Gudgeon | Omnivorous | Middle-lower | 3 | 39 | 16 | 3.5 |
| *Siniperca chuatsi* | Mandarin Fish | Carnivorous | Middle-upper | W | N/A | 22 | 7.5 |
| *Xenocypris argentea* | Yellowfin | Omnivorous | Bottom | W | N/A | 28-29 | 6.0 |
|  |  |  |  | 1 | 33-148 | 14-29 | 4.5-5.5 |
| Shrimps | | Omnivorous | River floor | 1 | <1.0- 4.0 | 5.5-8.0 | N/A |
|  |  |  |  | 2 | 1.0 | 6.0 | N/A |
|  |  |  |  | 3 | 4.0 | 7.0 | N/A |
|  |  |  |  | 4 | 2.0-7.0 | 6.0-8.5 | N/A |
| Crabs | | Omnivores | River floor | 3 | 5.0 | 5.5 | 6.0 |
|  |  |  |  | 4 | 10 | 3.0-7.0 | N/A |
| Snails | | Herbivorous | River floor | 4 | <1.0- 33 | 2.5-6.5 | 2.5-6.5 |

Table S.6. Feeding habits, habitats, weight, length, and width of the aquatic species investigated near Dabaoshan mine, China.

| **Predator** | | **Prey 1** | **Prey 2** | **Prey 3** | **Prey 4** | **Prey 5** | **Prey 6** |
| --- | --- | --- | --- | --- | --- | --- | --- |
| *Erythroculter ilishaeformis* | *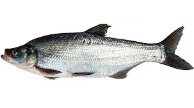* | *Carassius aumtus* |  |  |  |  |  |
| *Hemibarbus maculatus* | 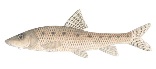 | snails | shrimps | crabs |  |  |  |
| *Pseudobagrus fulvidraco* | *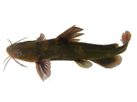* | *Carassius aumtus* | snails |  |  |  |  |
| *Siniperca chuatsi* | *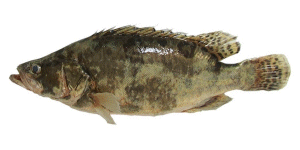* | *Carassius aumtus* | *Hemiculter leucisculus* | *Pseudorasbora parva* | *Cyprinus carpio* | *Sarcocheilichthys nigripinnis* | *Pseudobagrus fulvidraco* |

Table S.7. Carnivorous species and their corresponding preys collected from the Hengshi and Wengjiang Rivers near Dabaoshan mine, China.

Table S.8. Metal Pollution Index of aquatic species collected from the Hengshi and Wengjiang Rivers near Dabaoshan mine, China.

| Species | Site | Tissues | MPI |
| --- | --- | --- | --- |
| *A. rivularis* | 1 | Muscle | 0.44 |
| *A. macropterus* | W | Muscle | 0.21 |
|  |  | Gill | 1.89 |
|  |  | Intestine | 1.61 |
| *A. nobilis* | 4 | Muscle | 0.37 |
|  |  | Gill | 2.56 |
| *E. ilishaeformis* | 3 | Muscle | 0.48 |
| *H. maculatus* | W | Muscle | 0.01 |
|  |  | Gill | 0.75 |
|  |  | Intestine | 1.57 |
| *P. altivelis* | 2 | Muscle | 0.46 |
| *P. fulvidraco* | W | Muscle | 0.70 |
|  |  | Gill | 0.97 |
|  |  | Intestine | 18.1 |
| *P. parva* | 3 | Muscle | 0.48 |
| *S. nigripinnis* | 3 | Muscle | 0.59 |
| *S. chuatsi* | W | Muscle | 0.0 |
|  |  | Gill | 0.49 |
|  |  | Intestine | 0.08 |
| *H. bleekeri* | W | Muscle | 0.50 |
|  | 2 | Muscle | 0.51 |
| *H. leucisculus* | 4 | Muscle | 0.36 |
| *C. aumtus* | 1 | Muscle | 0.03 |
|  | 2 | Muscle | 0.59 |
|  | 4 | Muscle | 0.81 |
|  | W | Muscle | 0.42 |
|  |  | Gill | 0.87 |
|  |  | Intestine | 8.79 |
| *C. carpio* | 1 | Muscle | 0.39 |
|  |  | Gill | 1.55 |
|  |  | Intestine | 3.03 |
|  | W | Muscle | 0.02 |
|  |  | Gill | 1.19 |
|  |  | Intestine | 1.73 |
| *O. mossambcus* | 1 | Muscle | 1.06 |
|  |  | Gill | 0.65 |
|  | 2 | Muscle | 0.55 |
|  |  | Gill | 1.87 |
|  |  | Intestine | 8.47 |
|  | W | Muscle | 0.36 |
|  |  | Gill | 1.46 |
|  |  | Intestine | 27.0 |
| *X. argentea* | 1 | Muscle | 0.69 |
|  |  | Gill | 2.34 |
|  |  | Intestine | 44.0 |
|  | W | Muscle | 0.08 |
|  |  | Gill | 0.93 |
|  |  | Intestine | 18.9 |
| Shrimps | 1 | Muscle | 1.08 |
|  |  | Head | 5.56 |
|  | 2 | Muscle | 1.83 |
|  | 3 | Muscle | 0.91 |
|  |  | Head | 4.51 |
|  | 4 | Muscle | 1.26 |
|  |  | Head | 6.48 |
|  | W | Muscle | 1.93 |
|  |  | Head | 3.60 |
| Crab | 3 | Muscle | 2.84 |
|  | 4 | Muscle | 3.53 |
| Snails | 4 | Muscle | 1.96 |
|  |  | Intestine | 40.7 |


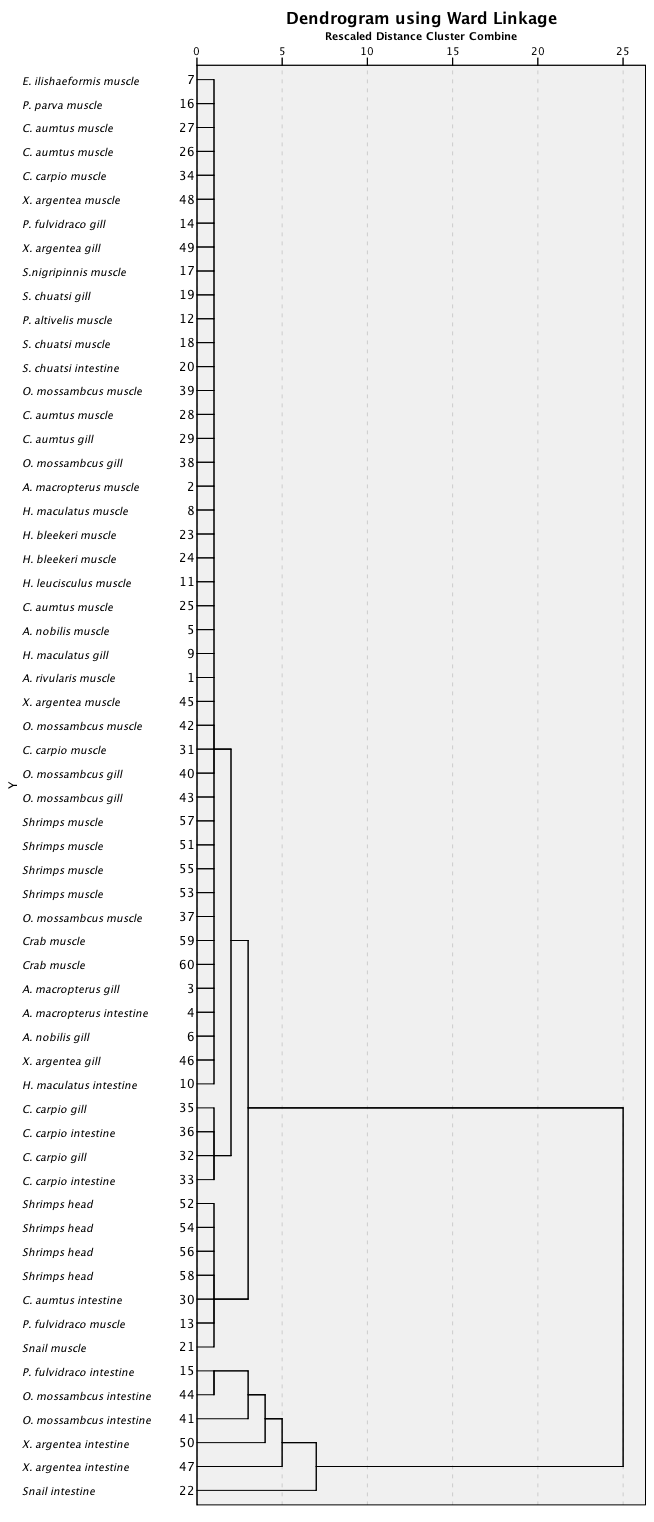


Figure S.1. Dendrogram of hierarchical clustering analysis of all studied aquatic organisms near Dabaoshan mine, China using Ward’s method.

Bugang, W. & Woolsey, M. 2012. National food safety standard-maximum levels of contaminants in food. *The* *People’s Republic of China. Gain Report Number-CH10000*.

China National Standards Management Department 2001. Safety qualification for agricultural product for non-environmental pollution aquatic products. Beijing, China.

China State Bureau of Quality and Technical Supervision 2002. The People’s Republic of China National Standards - Marine Sediment Quality. Beijing, China.

EPA, U. 2009. Toxicological Review of Thallium and Compounds.

European Commission 2005. Commission Regulation (EC) No 78/2005 of 19 January 2005 amending Regulation (EC) No 466/2001 as regards heavy metals.

Joint FAO/WHO Expert Committee on Food Additives 2010. *Evaluation of Certain Food Additives: Seventy-first Report of the Joint FAO/WHO Expert Committee on Food Additives*, World Health Organization.

UNEP 1985. Reference Methods for Marine Pollution Studies. *Determination of total Hg in marine sediments and suspended solids by cold vapour AAS.*

Wyse, E., Azemard, S. & Mora, S. 2003. World-wide intercomparison exercise for the determination of trace elements and methylmercury in fish homogenate IAEA-407. *IAEA Marine Environment Laboratory*.
